# Self-Organizing Feature Maps Identify Proteins Critical to Learning in a Mouse Model of Down Syndrome

**DOI:** 10.1371/journal.pone.0129126

**Published:** 2015-06-25

**Authors:** Clara Higuera, Katheleen J. Gardiner, Krzysztof J. Cios

**Affiliations:** 1 Departamento de Bioquímica y Biología Molecular I, Facultad de Ciencias Químicas, Universidad Complutense, Madrid, Spain; 2 Departamento de Inteligencia Artificial e Ingeniería del Software, Facultad de Informática, Universidad Complutense, Madrid, Spain; 3 Linda Crnic Institute for Down Syndrome, Department of Pediatrics, Department of Biochemistry and Molecular Genetics, Human Medical Genetics and Genomics, and Neuroscience Programs, University of Colorado, School of Medicine, Aurora, Colorado, United States of America; 4 Department of Computer Science, Virginia Commonwealth University, Richmond, Virginia, United States of America; 5 IITiS, Polish Academy of Sciences, Gliwice, Poland; IGBMC/ICS, FRANCE

## Abstract

Down syndrome (DS) is a chromosomal abnormality (trisomy of human chromosome 21) associated with intellectual disability and affecting approximately one in 1000 live births worldwide. The overexpression of genes encoded by the extra copy of a normal chromosome in DS is believed to be sufficient to perturb normal pathways and normal responses to stimulation, causing learning and memory deficits. In this work, we have designed a strategy based on the unsupervised clustering method, Self Organizing Maps (SOM), to identify biologically important differences in protein levels in mice exposed to context fear conditioning (CFC). We analyzed expression levels of 77 proteins obtained from normal genotype control mice and from their trisomic littermates (Ts65Dn) both with and without treatment with the drug memantine. Control mice learn successfully while the trisomic mice fail, unless they are first treated with the drug, which rescues their learning ability. The SOM approach identified reduced subsets of proteins predicted to make the most critical contributions to normal learning, to failed learning and rescued learning, and provides a visual representation of the data that allows the user to extract patterns that may underlie novel biological responses to the different kinds of learning and the response to memantine. Results suggest that the application of SOM to new experimental data sets of complex protein profiles can be used to identify common critical protein responses, which in turn may aid in identifying potentially more effective drug targets.

## Introduction

Down syndrome (DS) is the most common genetic cause of learning/memory deficits [1]. It is due to an extra copy of the long arm of human chromosome 21 (Hsa21) and the consequent increased level of expression, due to dosage, of some subset of the genes it encodes [2]. Although no pharmacotherapies for learning deficits in DS are available, because the incidence is high (one in 1000 live births worldwide [1]), there is considerable interest in their identification. DS is a genetic perturbation of considerable complexity. Hsa21 encodes >500 genes/gene models [3] and an unknown subset of these contribute to the learning deficits. Functional information is available for fewer than half of Hsa21 genes, and even for these information is limited. It is clear, however, that Hsa21 genes include transcription factors, protein modifiers (kinases, phosphatases, methylases and several involved in ubiquitination and sumoylation), RNA splicing factors/modifiers, cell surface receptors and adhesion molecules, and components of many biochemical pathways [4]. Overexpression of these genes, as is predicted to occur in DS, will perturb many different biological processes and pathways, including many affecting brain development and function [4]. Because of this complexity, for choosing drug targets, it is logical to consider the downstream, integrated, consequences of all Hsa21 genes that are overexpressed, i.e., to look for perturbations in pathways that are critical to learning and memory and then to consider drugs that would correct the observed perturbations. An advantage of a pathway investigation is that detailed understanding of the functions of individual Hsa21 genes, when and where each is overexpressed, and their specific interactions and contributions to brain function, is not needed.

For preclinical evaluation of drug effects, multiple mouse models of DS, each carrying an extra copy of a subset of Hsa21 orthologous genes, have been created (reviewed in [5]). In prior work, we have reported baseline pathway perturbations at the protein level in several of these [4, 6–9]. More recently, to investigate normal responses to learning, we measured levels of ~80 proteins in subcellular fractions in brain regions of wild type mice exposed to context fear conditioning [10], a task commonly used to assess associative learning [11]. In these mice, approximately half the proteins responded to learning in CFC in at least one fraction/brain region. We also examined the effects of memantine on protein expression, with and without learning in CFC. Memantine is currently in use for treatment of moderate to severe Alzheimer’s Disease (AD) [12] and has been proposed for treatment of learning deficits in DS [13,14]. While memantine is known to modulate excitatory neurotransmission through antagonizing activity of N-methyl-D-aspartate (NMDA) receptors [15], little is known about its effects on protein expression, either alone or with learning paradigms. Acute treatment with memantine does not affect learning in control mice [14] but does alter initial protein profiles and modulates molecular responses to CFC [10]. We have similarly analyzed protein responses in the partial trisomy mouse model of DS, Ts65Dn [16]. Untreated Ts65Dn mice fail to learn in CFC but if they are first injected with memantine, they learn successfully, i.e., learning is rescued [14]. Comparing protein profiles between the Ts65Dn when they fail in CFC, the Ts65Dn when their learning is rescued with memantine and similarly treated control mice revealed statistically significant changes in protein levels associated with normal, failed and rescued learning, changes in protein levels caused by memantine treatment alone, and differences in responses between control and Ts65Dn mice. The complexities of these data are novel and they provide a more realistic view of the molecular consequences of learning than is seen in the more common types of studies that examine effects of single gene mutations and measure the levels of fewer than 5–10 proteins. The standard statistical analyses employed in the Ts65Dn CFC studies [16] do not, however, identify several important features, for example, which of the changes seen in control mice are required for successful learning, which of the abnormalities in the Ts65Dn directly contribute to failed learning, and which changes induced by memantine are critical for rescuing successful learning in the Ts65Dn.

To begin to answer these types of questions, computational learning methods can be explored. Both supervised and unsupervised learning have been shown to be very useful in analysis of biological data [17, 18]. Supervised learning is used when data instances belong to known classes, i.e., each instance is associated with a class label. Supervised learning methods include rule based classifiers, Support Vector Machine (SVM), decision trees, and Bayesian classifiers. Their goal is to build a general model that can subsequently be used to classify new, unlabeled data instances.

In contrast to supervised learning, in unsupervised learning, the class labels of data instances are not known. The key unsupervised learning technique is clustering, which divides the data instances (each described by a set of features) into groups based on similarities in these features. Examples of unsupervised learning methods include Self-Organizing Feature Maps (SOM), Growing Cell Structures (GCS), K-means, and hierarchical clustering. Each has advantages and limitations. SOM consists of an artificial neural network algorithm that clusters multidimensional data and presents the result in a two dimensional (2-D) projection or map of nodes. Because data instances that are close in the input space are clustered in nodes that are also close in the 2-D map, SOM preserves the topology of the original multidimensional input data. Providing this visualization of the structure or clusters present in the data differentiates SOM from other clustering methods. A second important advantage of SOM is that the number of clusters in the data is not specified a priori. This is in contrast to, for example, the K-means algorithm; the disadvantage of specifying the number of clusters beforehand is that the data will be forced into that number of clusters regardless of the “true” number. Lastly, with SOM, because the data are grouped within a 2-D map, it is possible to identify groups of data, not observable in highly-dimensional input data, and to visualize the borders between the groups, which together can lead to identification of new patterns. SOM has proven to be a popular and powerful bioinformatics tool, in particular in analysis of gene expression data where the goal was to identify functional or regulatory similarities among genes with similar expression profiles [19–27].

GCS is a clustering method with similar properties to SOM. In it, nodes of the network are inserted and deleted automatically as needed [28]. However, tuning of the parameters (e.g., threshold of removal, **μ**) is not trivial because it depends on the nature of the data. Configuring the network for training also requires complex additional pre-processing to decide which values of the parameters work best for a given data set.

In this work, we applied SOM to expression data for 77 proteins (i.e., a 77-dimensional space) obtained from the nuclear-enriched fraction of cortex from control and Ts65Dn trisomic mice. We first show that SOM clusters mice that differ in genotype, treatment and success in learning into separate classes that reflect differences and similarities in protein expression levels. SOM successfully clustered and discriminated classes of control mice based on treatment with memantine, and with and without the stimulation to learn. It also discriminated failed learning in the Ts65Dn mice from rescued learning in the Ts65Dn and from normal learning in control mice. We also show that repeating clustering with subsets of the 77 proteins, identified using the Wilcoxon rank-sum test, generated SOMs with new class characteristics. These SOMs provided validation that data from smaller numbers of proteins can successfully discriminate between failed and normal learning and predict proteins that are most relevant to rescued learning.

These results suggest that SOM applied to additional datasets can help to identify those protein abnormalities in DS mice that most critically need to be altered by drug treatments to facilitate the rescue of learning impairments.

## Materials and Methods

### Protein samples and groups of mice

**Fig 1:**
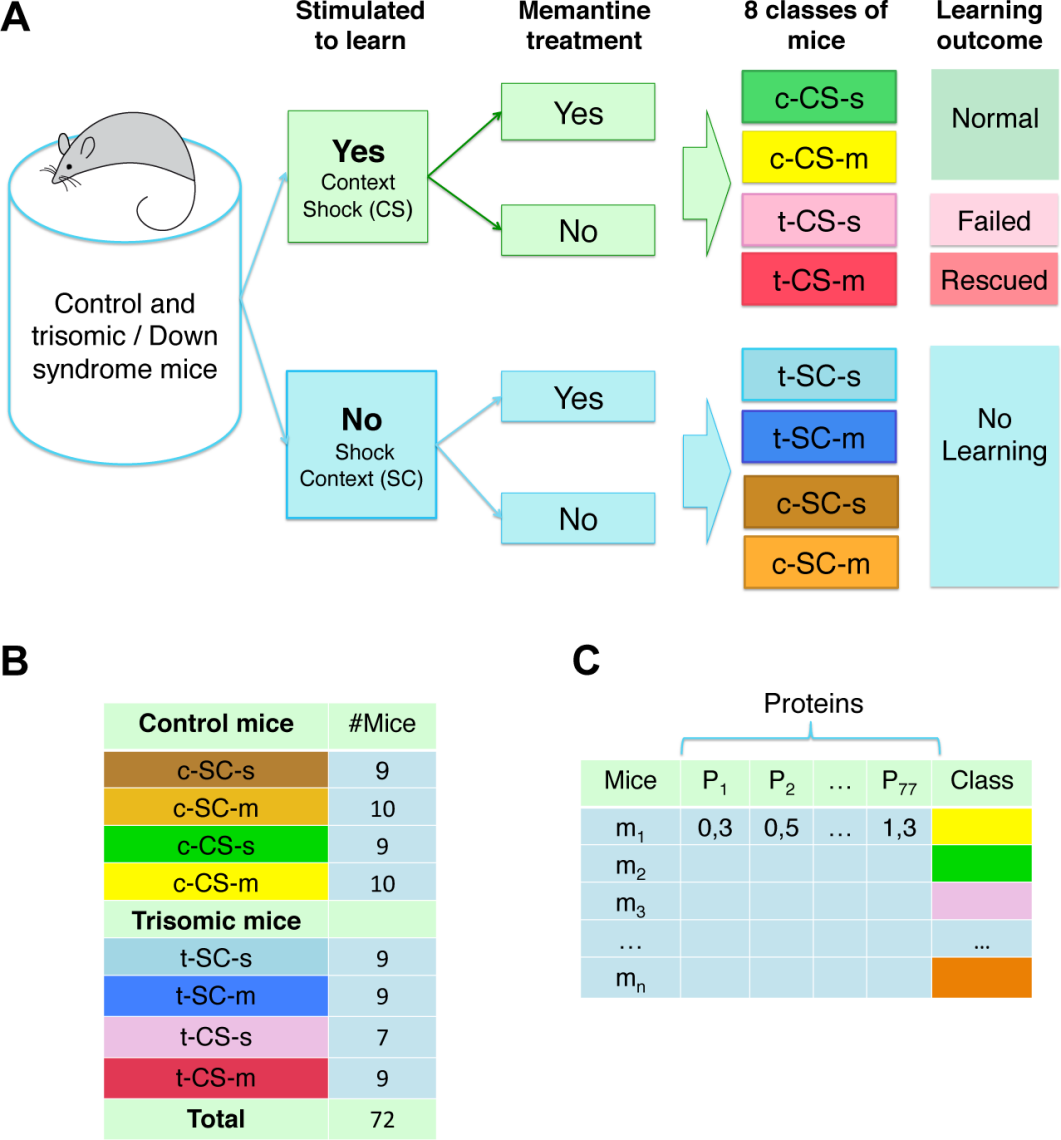
Classes of mice. **(A)** There are eight classes of mice based on genotype (control, c, and trisomy, t), stimulation to learn (Context-Shock, CS, and Shock-Context, SC) and treatment (saline, s, and memantine, m). Learning outcome indicates the response to learning for each class. **(B)** Number of mice in each class. **(C)** Format of protein expression data: rows are individual mice, and columns, P _1 …_ P_77,_ are the measured levels of the 77 proteins. The color coding in the last column identifies each class; color coding was used in visualization of the SOMs but not in clustering.

All mice, protein samples and protein expression levels have been reported previously [10,16]. Briefly, protein lysates were prepared from brains of 3 month old male Ts65Dn Down syndrome model mice and their male littermate wild type controls, after training in context fear conditioning (CFC) with and without injection with the drug memantine. The basic CFC protocol requires two groups of mice [29]. The context-shock (CS) group are placed in a novel cage, allowed to explore for several minutes and then given a brief electric shock; normal, wild type mice learn to associate the novel context with the aversive stimulus and will freeze upon re-exposure to the same cage. To control for the effects of the shock alone, a second group of mice, the shock-context (SC) group, are placed in the novel cage, immediately given the electric shock, and then allowed to explore; with these conditions, normal, wild type mice do not learn to associate the novel cage with the shock and do not freeze upon re-exposure to the same cage [29]. Unlike their wild type littermates, the Ts65Dn CS group of mice fail to learn and do not freeze; this learning impairment can be corrected, however, if the Ts65Dn are injected with memantine prior to training [14]. To control for the effects of injection alone, an additional CS group injected with saline (no drug) must be included and to control for effects of injection and memantine alone, separate SC groups must also be injected with saline and with memantine. Thus, with a drug treatment included in CFC, four groups of Ts65Dn were required: CS-saline, CS-memantine, SC-saline and SC-memantine. The same four groups of wild type control littermates were also required. Thus, a total of eight groups of mice were generated and protein expression levels were measured for each. Classes of mice, associated treatments and learning outcomes are listed in Fig 1A. The numbers of mice per group and the input data format are shown in Fig 1B and 1C, respectively.

### Dataset

The dataset used here (S1 Dataset) consists of the expression levels of 77 proteins/protein modifications that produced detectable signals in the nuclear fraction of cortex. There were 38 control mice and 34 trisomic mice, for a total of 72 mice (7–10 mice in each of the eight groups (Fig 1). Measurements were made using reverse phase protein arrays (RPPA), a high throughput technique in which protein samples from individual mice are robotically spotted onto nitrocellulose-coated microscope slides [30]. For experiments here, a single slide contained 20 spots per sample: three replicates of a five-point dilution series, plus replicate buffer controls, i.e., 15 measurements of each protein per sample. Therefore, for control mice, there were 38x15, or 570 measurements, and for trisomic mice, there were 34x15, or a total of 510 measurements, per protein. RPPA is highly sensitive and reproducible [7] but technical artifacts can occur which require the elimination of data from individual spots. Similar to other high throughput techniques, it is not possible to repeat experiments for individual measurements and therefore the final dataset contains missing values, i.e., there were <15 measurements for a small number of samples/proteins measured (see below). Protein expression data were generated from additional subcellular fractions from both cortex and hippocampus of the same mice. However, the cortex nuclear fraction was chosen for use here because it was the most complete of the datasets.

### Overview of analysis

SOM, also referred to as a Kohonen map, is an artificial neural network implementation of a clustering algorithm [31]. SOM was used here to cluster data from the eight classes of mice, without using the class information. SOM does not require the user to specify the number of clusters; by not forcing the data into a fixed number of clusters, the SOM can reveal correct or true cluster structure within the data. In addition, the input multidimensional data (here, 77 proteins or features) is projected onto a 2-D space or map of nodes, where each instance of the data is clustered into a specific node. This 2-D presentation facilitates interpretation of results. In the computational literature, the processing elements of an SOM are referred to as “artificial neurons”. However, to prevent confusion between the use of “neurons” to refer to specific cells within the brain involved in learning and “artificial neurons” that are the building blocks of any artificial neural network, including the SOM, here we use the term node as equivalent to and meaning "artificial neuron".

Data analysis consisted of four steps: 1) preprocessing of the data, 2) determining the optimal size of the SOM, clustering and labeling, 3) identifying class-specific clusters and using the Wilcoxon Rank Sum test to identify proteins that discriminate between classes, and 4) validating the results by repeating SOM clustering using as input, data only from subsets of proteins, i.e., using the same number of mice (data instances) but reducing the dimensionality. Note that information of the mouse classes was not used by SOM algorithm; it was used only later for labeling of the clusters identified.

#### 1. Data preprocessing

Data for seven of 77 proteins had one or more mice with missing values. Instead of removing these proteins, we replaced the missing values with the average value of the expression level of that protein in the same class of mice. One mouse in the t-CS-m (trisomy-CS-memantine) group had missing values for the majority of proteins and values that were very different from other mice of its same class. We therefore considered this mouse an outlier and excluded it from further analysis.

SOM requires that all input features (protein expression levels) have a similar range of values because of the following. If some proteins have values in the range 0–3 and others between 0 and 0.6 (as is the case in our data), then the proteins with higher values would have more influence on the clustering outcome, possibly leading to erroneous results. Thus all measurements (a matrix where samples/mice measurements are the rows and proteins are the columns) are normalized to the range 0–1, column by column, using Eq 1.

$$Normalized\left(e_{ij}\right)=\;\left(e_{ij}-E_{j,min}\right)/\;\left(E_{j,max}-E_{j,min}\right)$$ (1)

where e_ij_ is the value of expression level of mouse i for protein j. *E*_*j*,*min*_ and *E*_*j*,*max*_ the minimum and maximum of all the values of protein j (min and max of the column).

#### 2. Determination of the SOM size, clustering and labeling

SOM allows visualization of the clustering result in the 2-D space. Nodes in the SOM are arranged in a grid of *n* rows by *n* columns. Because SOM preserves the topology of the original data in the projected 2-D space, it groups data items within nodes so that those close in the original space are also close in the projected 2-D space. Here, mice are clustered in nodes according to similarities in their protein levels. Hence, if protein levels differentiate between classes, we expect to find two or more zones on the map, e.g., one zone containing c-CS-s (control-CS-saline) mice that learn and another containing c-SC-s (control-SC-saline) mice that do not learn.

Application of SOM requires estimation of its size, i.e., deciding the number of nodes into which the input data are to be clustered. We selected the number of nodes such that data from a single mouse (all 15 measurements) could be clustered within a single node. This assured that if there were no clustering structure present in the data, no artificial grouping of mice would be imposed (which might be the case if the number of nodes was too small). Thus, for 570 measurements (per protein) corresponding to 38 control mice, the SOM size chosen was 7x7. A smaller size (e.g., 6x6 or 5x5) might overly compact the data, possibly forcing clustering of measurements from more than one class of mouse into a single node. A larger size (e.g., 8x8 or 9x9) might prevent identification of true clusters. For the 510 measurements (per protein) corresponding to the 34 trisomic mice, a SOM size of 6x6 was chosen.

SOM was implemented using the neural network toolbox of Matlab (R2011b). SOM starts by assigning random weights to the nodes. Consequently, different runs produce slightly different clustering results. To choose the best SOM, we use the average quantization error, ε_q,_ defined in Eq 2. It measures how well the data samples are adapted to the resulting map. m_c_ is the weight vector of each node, x_i_ is a data sample clustered in a node (in our case, protein measurements) and N is the number of nodes. The lower the value of ε_q_, the closer the data elements are to their best matching node. The value of ε_q_ was calculated for ten runs and the SOM with the minimum value of ε_q_ was selected.

$$\epsilon_{q}=\frac{1}{N}\sum_{i=1}^{N}\left|\left|x_{i}-m_{c}\right|\right|$$ (2)

Ideally, mice from different classes would be separated into different clusters (represented by groups of nodes of the SOM map). If SOMs from different runs produced identical minimum values of the average quantization error, then the SOM with the smallest number of nodes that grouped mice from different classes was selected.

Visualizing clustering in the 2-D space of the SOM facilitates biological interpretation. We further automatically color-coded each node according to the majority class of its content and labeled each with the name of the majority class (of mice clustered in it). Note that class information is not used during SOM clustering but only when labeling the nodes for visualization.

#### 3. Identification of class-specific clusters and discriminant proteins

The distribution of the classes of mice (Fig 1A and 1B) within the final SOM map was used to evaluate the validity of the approach. If the SOM clusters the majority of mice from the same class in one or several adjacent nodes, thus forming large class-specific clusters, this suggests that the protein levels (features) discriminate well between classes. For our purpose, we define a cluster as: (i) two or more adjacent nodes that contain mice of the same class and no mice from other classes, or (ii) a single node that contains ≥ 80% (or ≥12 of 15) of the measurements of one mouse and no measurements of mice from any other class. For identifying clusters, we disregard nodes that group mice from different classes.

Each class of mice (represented by a group of nodes) is associated with a set of weight vectors. The weight vector of a node is a vector of the same dimension (i.e., 77) as the input data and represents the mice clustered in it.

To identify proteins whose levels are significantly different between classes we use the Wilcoxon rank-sum test, often used in gene expression analysis [32–36]. Here, this test was used to compare the weight vectors of the nodes defining clusters of two classes. For example, if Class A mice are found in one large cluster, C_A_, composed of 15 nodes, and Class B mice are found in another large cluster, C_B_, composed of 12 nodes, and P_1_ is a protein of interest, then a set of values C_A_P_1_ contains the 15 values of the weight vectors of C_A_ for P_1_ and a set of values, C_B_P_1,_ contains the 12 values of the weight vectors of C_B_ for P_1_. The Wilcoxon test was run 77 times (for values of each protein) for comparing Classes A and B as follows:

$$\begin{array}{l} \text{Wilcoxon}\_\text{test}\;\left({\text{C}}_{1}{\text{P}}_{1},\;{\text{C}}_{2}{\text{P}}_{1}\right)\;=0.001\\ \text{Wilcoxon}\_\text{test}\;\left({\text{C}}_{1}{\text{P}}_{2},\;{\text{C}}_{2}{\text{P}}_{2}\right)=0.3\\ .\\ .\\ .\\ \text{Wilcoxon}\_\text{test}\;\left({\text{C}}_{1}{\text{P}}_{77},\;{\text{C}}_{2}{\text{P}}_{77}\right)=0.002 \end{array}$$

The Wilcoxon test returns a p-value; proteins with p < 0.05 were considered to be significantly different between the two classes and the set of such proteins therefore discriminates between the two classes. Weight vectors are used because they represent characteristics of the measurements clustered in each node, retaining the most characteristic values of expression levels of each class. Given the eight classes of mice, there are 28 possible pairwise comparisons but only a subset of these are biologically meaningful. Table 1 lists the comparisons performed and their biological relevance.

**Table 1:** Group comparisons and biological relevance.

Groups	Biological interpretation
c-CS-s vs. c-SC-s	Effects of CFC training in saline injected controls (normal learning, NL)
c-CS-m vs. c-CS-m	Effects of CFC training in memantine injected controls (normal learning, NLm)
c-SC-m vs. c-SC-s	Effects of memantine on control baseline
c-CS-m vs. CS-s	Effects of memantine on control final conditions (normal learning +/- memantine)
t-CS-s vs. t-SC-s	Effects of CFC training in saline injected Ts65Dn (failed learning, FL)
t-CS-m vs t-SC-m	Effects of CFC training in memantine-injected Ts65Dn (rescued learning, RL)
t-SC-m vs. t-SC-s	Effects of memantine on trisomy baseline
t-CS-m vs. t-CS-s	Effects of memantine on Ts65Dn final conditions (RL vs. FL)
t-SC-s vs. c-SC-s	Initial trisomy vs. control differences

#### 4. Validation of results by repeating SOM clustering with different subsets of proteins

Once discriminant proteins were identified between classes, additional SOM clusterings were generated by including or excluding discriminant proteins. If the reduced subsets of proteins are indeed critical to class discrimination, then in the former, the quality of the clustering should improve (or remain the same) and in the latter, it should deteriorate. To identify proteins that are critical to learning, we further identified the intersection between different subsets of proteins that discriminated successful learning in control mice with and without memantine and failed vs. rescued learning in the Ts65Dn mice.

#### 5. Pathway enrichment

All proteins used in these experiments were selected because of their known roles in brain development, structure or function, and/or because abnormalities have been observed in brains from individuals with intellectual disability, Down syndrome, or Alzheimer’s Disease, or associated mouse models. To further assess relationships among proteins, the subsets that discriminated between different classes of mice were used to search the Kyoto Encyclopedia of Genes and Genomes Database (KEGG http://www.genome.jp/kegg/ [37]). Pathways that included among their components one or more discriminant proteins were identified, and all components of these pathways were retrieved. Protein interaction partners of each pathway protein component were obtained from the IntACT (http://www.ebi.ac.uk/intact/) [38], HPRD (Human Protein Reference Database, http://www.hprd.org/) [39] and BioGRID (Biological General Repository for Interaction Datasets, http://thebiogrid.org/) [40] databases; all interacting proteins that were also discriminant proteins were retained. Each pathway was annotated with the number and percent of components and the number and percent of interacting proteins that were also discriminant proteins.

## Results

SOM was used to cluster protein expression data from the eight classes of mice (four classes each of control and Ts65Dn mice). For both control and Ts65Dn, two groups of mice were trained in CFC (CS mice), injected with either saline or memantine, and two groups (SC mice) were not trained in CFC (i.e. were not stimulated to learn), also injected either with saline or memantine. The Ts65Dn CS mice injected with saline fail to learn the CFC task, but if injected with memantine, they learn successfully, while control CS mice learn equally well with either saline or memantine [14]. Levels of 77 proteins were quantitated by Reverse Phase Protein Arrays. In prior work, using a standard three-level mixed effects model plus a Bonferroni correction, it was demonstrated that levels of many of the 77 proteins differed between classes of mice due to genotype, training or injection [10, 16].

Questions of biological interest include: (i) can SOM correctly cluster mice into classes based on patterns of expression of the 77 proteins, (ii) can the resulting clusters and class separations be improved using subsets of the 77 proteins, and (iii) can subsets of proteins that are most critical for normal, failed and rescued learning, and memantine response be identified? We applied SOM first to the four classes of control mice to investigate its performance with protein profiles associated with normal learning. We then applied it to trisomic mice to investigate failed and rescued learning, and lastly, we applied it to a mix of control and trisomic mice to investigate patterns most relevant to learning impairment.

### 1. SOM clustering and labeling of data from control mice

Because the data are composed of 570 measurements (38 mice x 15 measurements per mouse), a SOM of the size 7x7 was selected. The SOM algorithm was run ten times. Table 2 shows the average quantization error, the number of mixed-class nodes, and the total number of measurements in mixed-class nodes for all runs. Run 4 (in bold) has the lowest average quantization error, the lowest number of mixed class nodes, and minimum number of mice in those nodes; therefore this SOM was selected.

**Table 2:** Features of different SOM runs for the four classes of control mice.

Run # (SOM 7x7)	Average quantization error	# of mixed class neurons	Total # of measurements in mixed class neurons
1	0,589	10	144
2	0,585	10	140
3	0,583	9	131
**4**	**0,579**	**8**	**110**
5	0,591	10	153
6	0,592	11	158
7	0,581	8	126
8	0,586	9	142
9	0,594	10	143
10	0,596	9	129

Bold indicates the best result.

**Fig 2:**
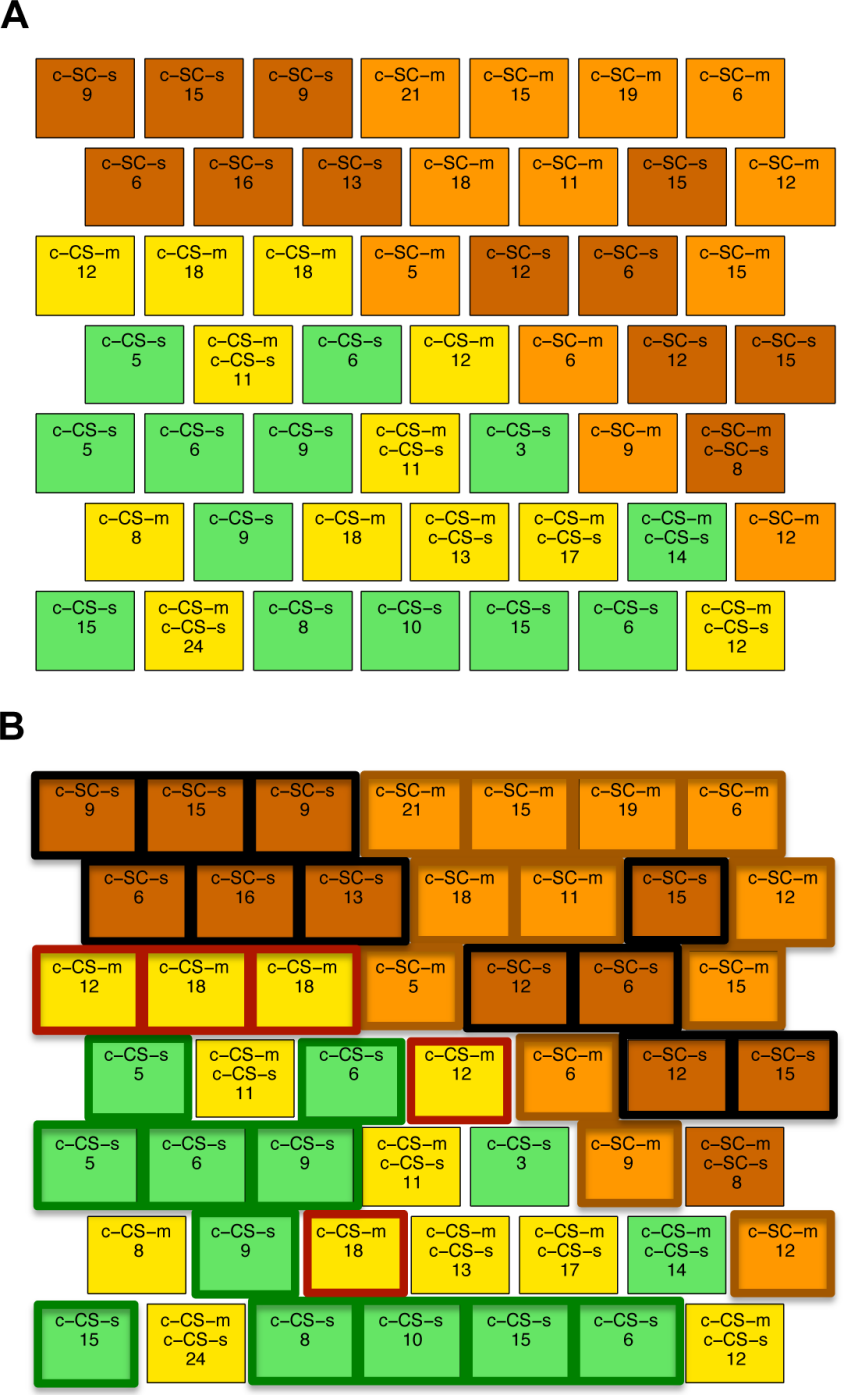
(A) SOM clustering of four classes of control mice. Node color indicates the majority class of the measurements clustered within it: brown: c-SC-s; orange: c-SC-m; green: c-CS-s, yelow: c-CS-m. Nodes are labeled with the name(s) of the majority and minority class(es) and the total number of measurements contained within them is also shown. (B) Strong class-specific clusters are outlined as black: c-SC-s; brown: c-SC-m; green: c-CS-s; dark red: c-CS-m.

Measurements from all mice were clustered in nodes in the 2-D SOM map (Fig 2A). We used a hexagonal layout where each node (represented by a square) has six adjacent nodes, with the exception of nodes on the outside borders. Because SOM preserves the topology of the original data, the data elements (measurements for each mouse) grouped within adjacent nodes correspond to measurements that are close to each other in the original 77-dimensional protein expression input space. After clustering, each node was labeled with a color representing the majority class gathered in it, the name(s) of the majority and minority class(es) and the total number of measurements contained within it. The two classes of control mice that learn successfully (c-CS-s and c-CS-m, green and yellow) are clearly separated from the two SC classes that do not learn (brown and orange). This suggests that learning in control mice is associated with distinct differences in protein expression levels. Fig 2B shows the same SOM with class-specific clusters outlined, where a cluster is defined either by a group of adjacent nodes that contain measurements from the same single class of mice or by a single node that contains a high percentage of measurements of the same type of mouse (recall that, here, mixed class nodes are not considered as valid cluster members). Among the two SC classes, saline treated mice (c-SC-s) form two large clusters of six and five nodes each (nodes outlined in black in Fig 2B) and memantine treated mice (c-SC-m) form one large cluster of nine nodes and two additional clusters of two and a single node each (nodes surrounded by brown line in Fig 2B). These clusters, plus the observation that only a single node mixes both c-SC-s and c-SC-m measurements, suggest that memantine alone is also associated with distinct differences in protein levels that discriminate memantine injection from saline. In contrast, the two CS classes are not so well separated into c-CS-s and c-CS-m clusters. While c-CS-s contains one large cluster of ten nodes plus a singleton (nodes surrounded in green, containing 70% of the measurements in this class), the c-CS-m class is represented by a three node cluster and two single nodes that together contain only ~50% of the measurements (nodes surrounded by dark red line). Seven of the 25 CS nodes mix c-CS-s and c-CS-m. Memantine does not alter the success of learning and therefore the similarities in differences in protein levels in these CS nodes may predominantly reflect responses to learning not effects of memantine.

#### i) Control mice class-discriminant proteins

The Wilcoxon rank-sum test was used to compare levels of individual proteins between pairs of classes. Comparing c-CS-s vs. c-SC-s identifies proteins that differ after normal successful learning in CFC compared with no stimulation to learn. Thirty-one proteins were significantly different (S1 Table, column c1). Because these results were obtained using weight vectors, we also used the Wilcoxon test for the 31 proteins using the original values of proteins without normalization; this did not change the significance of the results (data not shown). Box plots of the values of c-SC-s and c-CS-s clusters for the 12 proteins with lowest p-values are shown in S1 Fig. Values in c-CS-s are greater than those in c-SC-s for all of these proteins, except SOD1, IL1B and ubiquitin. Using a three-level mixed effects model, followed by a Bonferroni correction [10], showed that 24 of these 31 proteins were similarly significantly different, with only a single additional protein that was significantly different in [10] but not in our analysis.

Successful learning is also reflected in the comparison of c-CS-m vs c-SC-m, where levels after normal learning have been modulated by pre-treatment with memantine. Twenty-three proteins were found to be significantly different (S1 Table, column c2). Again, this list is consistent with prior results [10], where all but one of these proteins were also found to be significantly different.

Comparing the lists of proteins significantly different after successful learning with saline and with memantine identified 18 proteins in common (S1 Table, bold, columns c1 and c2). These included BRAF, ERK and pERK, components of the MAPK signaling pathway that is well established to be critical to learning [41]. Also of note are the immediate early gene protein, EGR1 and the brain-derived nerve growth factor protein, BDNF that are also relevant to learning [42,43]. Given the interest of this study to DS, it is also notable that four Hsa21 proteins, APP, DYRK1A, ITSN1 and SOD1 are included as responding in both.

**Fig 3:**
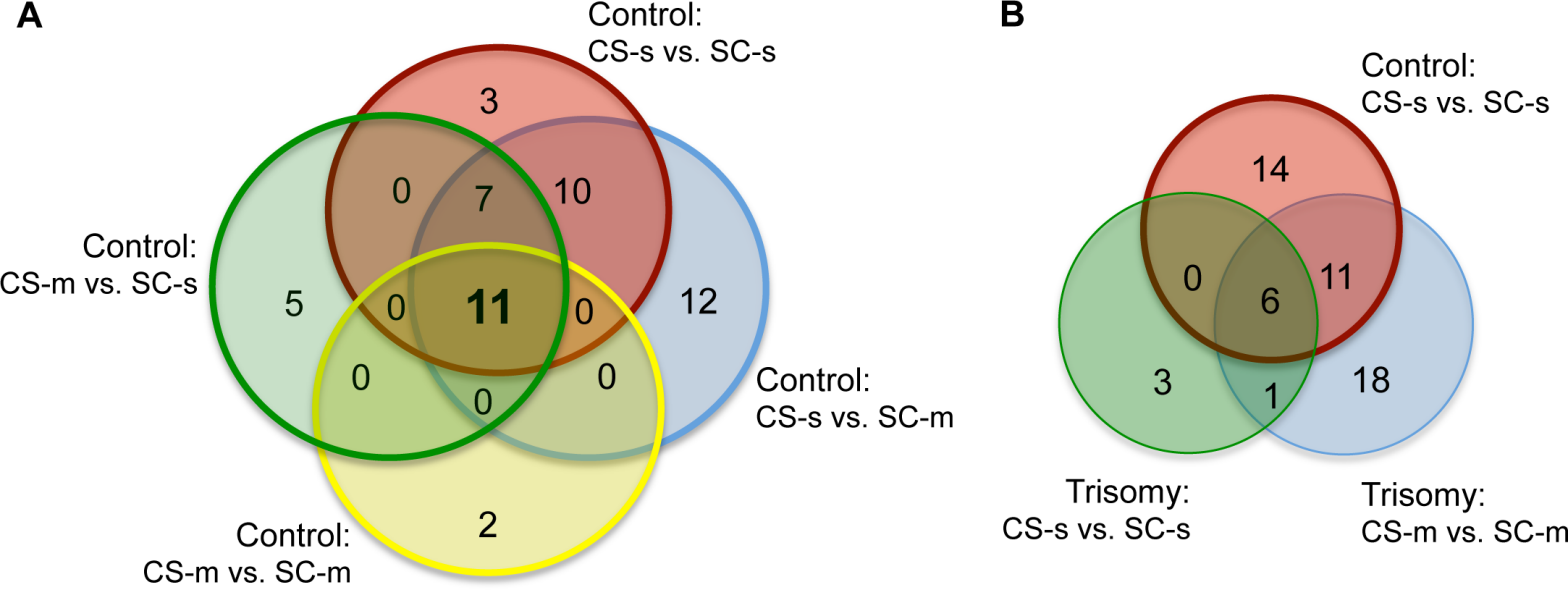
Venn diagrams of discriminating proteins. (A) Intersection of proteins discriminating learning in control mice. Colors indicate the proteins that respond in the four comparisons that reflect successful learning. (B) Intersection of proteins discriminating between normal, failed and rescued learning. Red circle: number of discriminant proteins between CS-s and SC-s in control (Normal learning; repeated from panel A). Blue circle: number of proteins that respond when learning is rescued in trisomic mice with memantine (CS-m vs. SC-m). Green circle: number of discriminant proteins in comparison CS-s vs. SC-s in trisomy (Failed learning).

Two additional comparisons reflect molecular events related to successful learning in control mice: c-CS-m vs. c-SC-s and c-CS-s vs. c-SC-m. Discriminant proteins in those two comparisons were identified (S1 Table, c3 and c4). Taking the intersection of all four successful learning comparisons identified 11 proteins: BRAF, CaNA, CDK5, DYRK1A, GFAP, ITSN1, pERK, pGSK3B, pNUMB, S6, and SOD1 (Table 3 column 1). In Fig 3A, a Venn diagram summarizes the numbers of proteins that respond in common or differentially in one or more of these four comparisons.

**Table 3:** Functional associations of discriminant proteins used to generate SOMs.

	1	2	3	4	5	6	7	8
**SOM**	Fig 4A	Fig 5B	Fig 5A	Fig 7A and 7B	Fig 7A	Fig 7B	Fig 8	Fig 9B
**Classes compared**	c-CS-s,m vs c-SC-s,m	c-SC-m vs c-SC-s	c-CS-m vs c-CS-s	t-CS-m vs t-SC-s,m	t-SC-m vs t-SC-s	t-CS-m vs t-CS-s	t-CS-s vs c-CS-s,m	t-SC-s vs t-SC-m, c-SC-s,m
**Function**	NL	MEM	MEM	RL	MEM	MEM	FL vs NL	
**MAPK**	BRAF	pCAMKII	PKCA	BRAF		BRAF		pRSK
	pERK		pPKCAB	pERK	pPKCAB	pERK		pPKCAB
		pPKCG		pMEK	pPKCG			pPKCG
		ELK		P38			P38	P38
**MTOR**				MTOR	pMTOR		MTOR	pMTOR
	S6	pS6	pS6		S6, pS6		S6	S6, pS6
	pGSK3BS9	AKT		AKT			RAPTOR	pGSK3BS9, T216
					pP70S6			pP70S6
**AD**	CANA			CANA				CANA
	CDK5					CDK5		CDK5
					P3525	P3525		P3525
			ERBB4		ERBB4		TAU	TAU
				SNCA	SNCA			SNCA
			IL1B					IL1B
			nNOS					
**NMDAR**		pNR1			pNR2A		pNR1	pNR2A
		NR1					NR2B	
	pNUMB	pNUMB				GLUR3	GLUR3	
**HSA21**	DYRK1A			DYRK1A		DYRK1A		
	ITSN1			ITSN1				
	SOD1	SOD1		SOD1		RRP1		
							APP	APP
**IEG**		ARC	ARC		ARC			ARC
			EGR1	EGR1			EGR1	
**Apoptosis**		BCL2	BAD		BAD			
**Histone**		H3AcK18		H3AcK18				H3AcK9
			H3MeK4	H3MeK4				
**Misc**	GFAP		SHH			GFAP		
		UBC	UBC	UBC		UBC		UBC

Figures illustrating each SOM and the classes compared in each are indicated. Where more than two classes were compared (columns 1, 4, 7 and 8), only those proteins common to all comparisons are listed; complete lists of proteins for each comparison are provided in S1 and S2 Tables. MAPK, components of the MAP kinase pathway; MTOR, components of the mechanistic target of rapamycin pathway; AD, proteins observed to be abnormal in brains from patients with or mouse models of Alzheimer’s Disease; NMDAR, subunits of ionotropic glutamate receptors and interacting proteins; Hsa21, proteins encoded by human chromosomes 21; IEG, immediate early gene proteins; apoptosis-related, BAD, proapoptotic, BCL2, antiapoptotic; histone, histone protein H3 modifications: Ac, acetylation, Me, methylation, K amino acid number of modified lysine residue; Misc, miscellaneous.

Also shown in Table 3 are the 13 proteins that discriminate c-SC-m vs. c-SC-s, indicating the effects of memantine on the initial conditions, and the 12 proteins that discriminate c-CS-m vs. c-CS-s, identifying the effects of memantine on the final protein levels after learning. Memantine is an antagonist of the N-methyl-D-aspartate receptor (NMDAR), and it is interesting to note that among memantine effects are the NMDAR subunit NR1 and its phosphorylated form, pNR1, plus phosphorylated forms of two proteins, NUMB and CAMKII, known to interact with and modulate the activity of and signaling from the NMDAR.

#### ii) Validation of discriminant protein subsets in control mice

The SOM in Fig 2 was generated using data from the complete set of 77 proteins. If the subsets of proteins found to discriminate class-specific clusters in this SOM are correct, then it should be possible to predict the outcome of the clustering when the input is limited to only those subsets.

**Fig 4:**
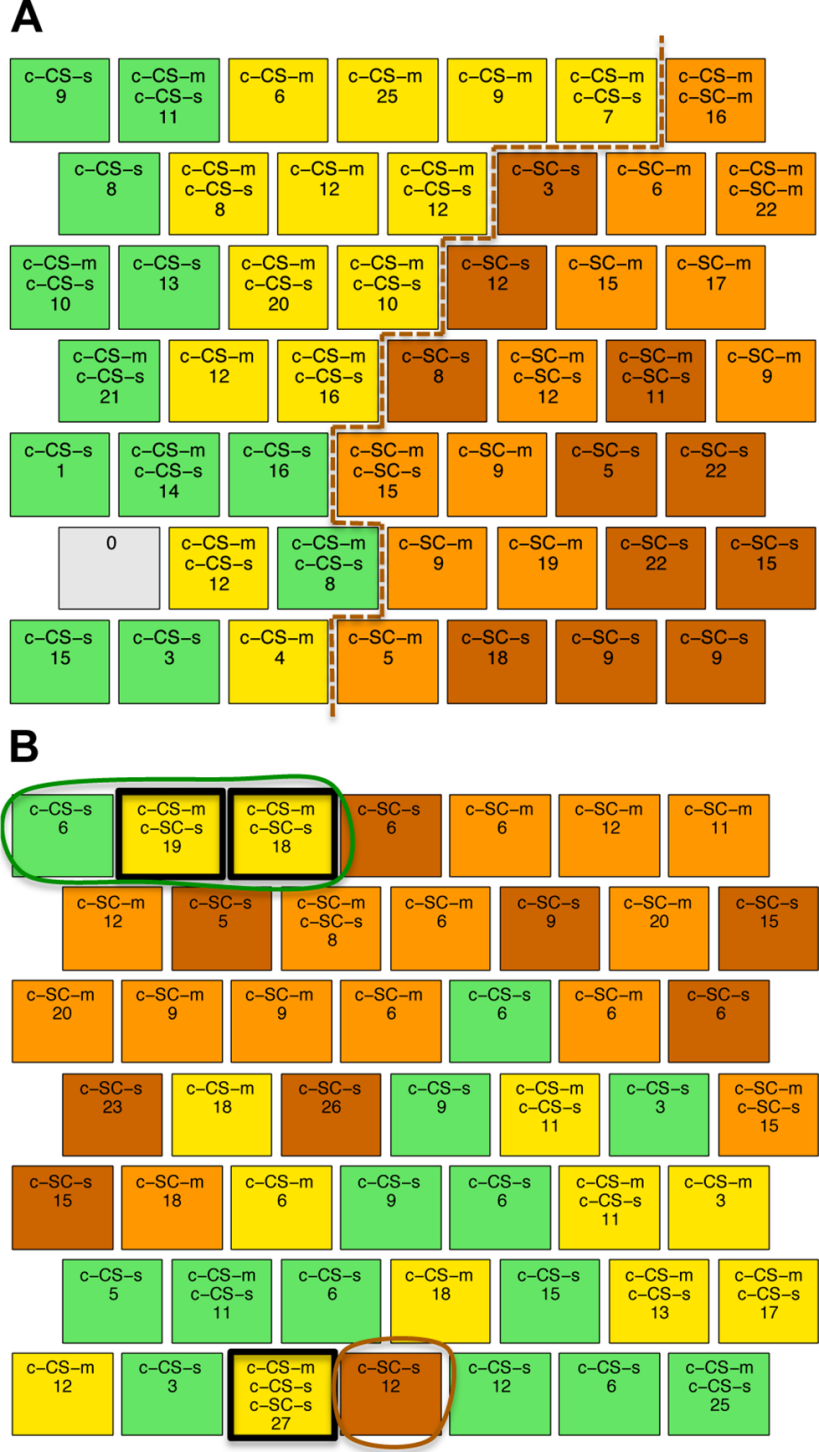
SOM clustering with subsets of protein expressions data from control mice. **(A)** SOM obtained using only 11 proteins (out of 77) that discriminate between the four classes of c-CS and c-SC. The dashed line indicates the border between the two main classes. **(B)** SOM obtained using the remaining 66 proteins. Outlined in green are nodes that contain a majority of CS mice surrounded by nodes with SC mice. Outlined in brown are nodes with SC mice surrounded by nodes of CS mice.

Fig 4A shows the SOM obtained using as input the 11 proteins found to be significantly different in all four comparisons related to successful learning (S1 Table, columns c1-c4; Table 3, column 1). The CS classes (yellow and green nodes) are clearly separated from the SC classes (brown and orange), indicating that proteins in this reduced subset are relevant to learning and also sufficient among the set of 77 to achieve separation. There are however differences between the qualitative features of the SOM in Fig 4A and that in Fig 2. In Fig 4A, there are 12 CS mixed nodes and they contain >50% of the measurements. This compares to only 7 CS mixed nodes containing <30% of measurements in the original SOM in Fig 2. This suggests that these 11 proteins are more relevant to identifying successful learning and less relevant to discriminating memantine responses.

Fig 4B shows the SOM generated with the remaining 66 proteins that were not common among the four successful learning classes. In contrast to the SOMs using the complete set of 77 and the reduced set of 11, the CS and SC groups are no longer well separated. Notably three CS (green and yellow) nodes are found in the upper left corner of the SOM, separated from the bulk of the CS nodes entirely. Two of them also contain measurements of SC mice. Similarly, there is one isolated SC node located within the bulk of the CS group (brown node in the bottom row) and another node contains measurements from three classes, c-CS-s, c-CS-m and c-SC-s mice. These results are important because they indicate the critical role of the 11 common proteins: without them the discriminating clusters are lost.

**Fig 5:**
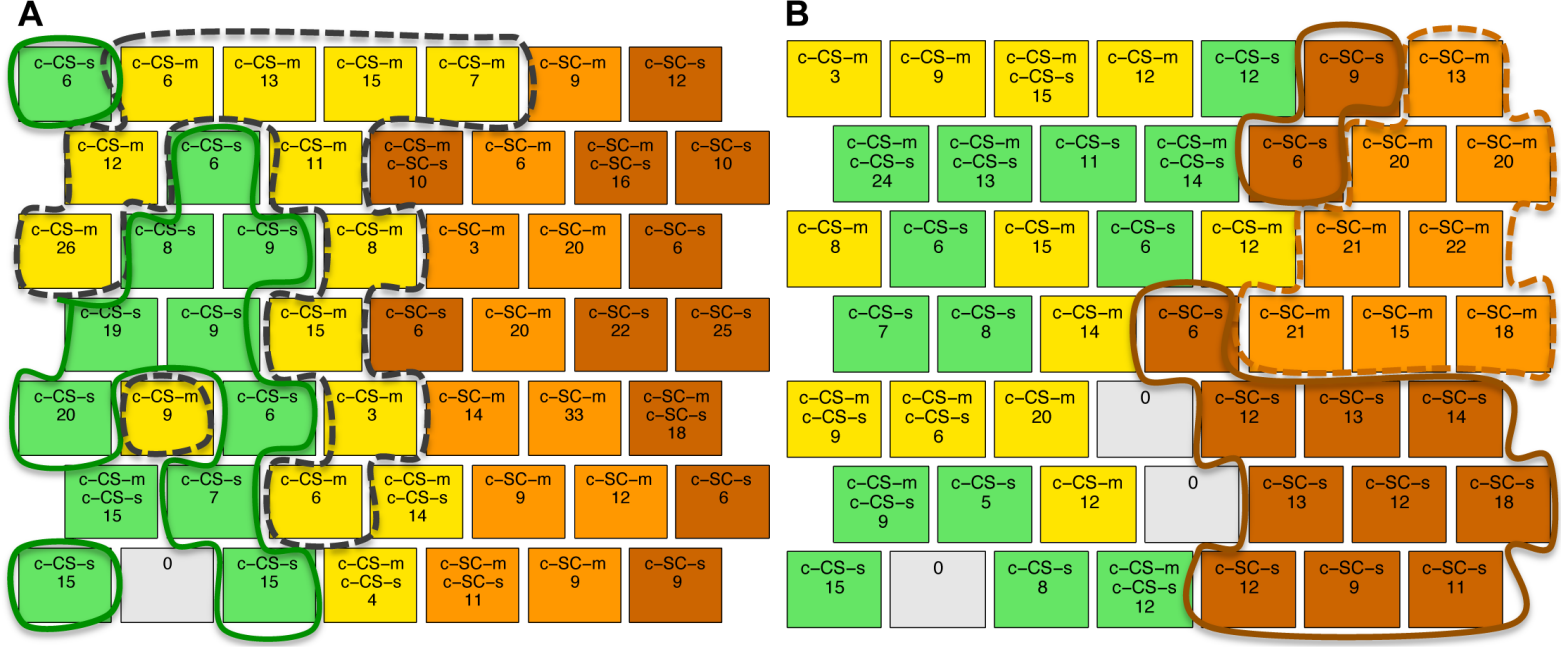
SOM clustering with data from the 11 proteins that discriminate between context-shock and shock-context (c-CS and c-SC) plus. (A) the 12 proteins that discriminate between context-shock with and without memantine (c-CS-m and c-CS-s). Dashed black line, clusters of CS-m; green outline, clusters of CS-s. (B) the 13 proteins that discriminate between c-SC-m and c-SC-s. Brown outline: c-SC-s clusters; orange dashed outline: c-SC-m cluster.

To further explore the effect of memantine on learning-induced protein expression, clustering was repeated using the 11 proteins common to the four c-CS-c-SC combinations from Fig 4A plus the 12 proteins that discriminate c-CS-s and c-CS-m classes, i.e. endpoint protein expression with and without memantine (Table 3 columns 1 and 3). The SOM is shown in Fig 5A. While the separation between CS and SC clusters remains clear, the separation between c-CS-s and c-CS-m has improved compared with previous SOMs. Specifically, the c-CS-s nodes form one large 9-node cluster plus one isolated node and the c-CS-m form a single 11-node cluster plus one isolated node. There are now only three mixed CS nodes and these contain only 33 measurements, a considerable reduction from the 12 mixed nodes containing 142 measurements when the 12 c-CS-s vs c-CS-m discriminating proteins were not included. These results suggest that the 12 proteins predominantly reflect memantine effects, not responses to learning. Table 4 summarizes the number of mixed c-CS-m and c-CS-s nodes in SOMs from Figs 2, 4A and 5A.

**Table 4:** Numbers of mixed c-CS-m and c-CS-s nodes and measurements.

# proteins used in clustering	77	11 (discriminating c-CS vs. c-SC)	11+12 (discriminating c-CS-m vs. c-CS-s)
**Reference figure**	Fig 1	Fig 2A	Fig 3B
**# of nodes with mixed c-CS-m and c-CS-s**	7	10	3
**# of measurements in mixed nodes**	102	142	**33**

Lastly, we added the 13 proteins that describe memantine effects on the initial protein profiles, c-SC-m vs c-SC-s, to the 11 that discriminate successful learning (Fig 5B) (the total number of proteins is actually 22 not 24 because SOD1 and pNUMB are present in both groups of proteins). The SOM result revealed that the c-CS and c-SC mice remained clearly separated, but the separation between c-SC-s and c-SC-m was improved. The clusters are completely separated, with c-SC-s measurements in two clusters composed of 10 and 2 nodes and c-SC-m measurements entirely contained in a single 8 node cluster. In addition, there were no mixed nodes. However, for c-CS-s and c-CS-m nodes, there are now more mixed neurons. This reflects the fact that some of the proteins that discriminate between c-SC-m and c-SC-s have similar values in, and do not discriminate between, c-CS-m and c-CS-s.

### 2. SOM clustering and labeling of data from trisomic mice

The four classes of trisomic mice, SC and CS with saline and memantine treatment, comprised a total of 34 mice and 510 measurements. A 6X6 SOM size was selected. Table 5 shows the average quantization error, the number of mixed class nodes and the total number of mice in mixed class nodes for ten runs of SOM clustering. Run 5 (bold) has the lowest values of all three measures.

**Table 5:** Features of different SOM runs for the four classes of trisomic mice.

Run SOM 6x6	Average quantization error	# mixed class neurons	Total # of measurements in mixed class neurons
1	0,7	9	150
2	0,7019	7	110
3	0,7014	8	148
4	0,7053	9	150
**5**	**0,6981**	**5**	**84**
6	0,7015	8	115
7	0,7006	6	92
8	0,7003	7	137
9	0,7053	7	116
10	0,7181	11	191

Bold indicates the best result.

**Fig 6:**
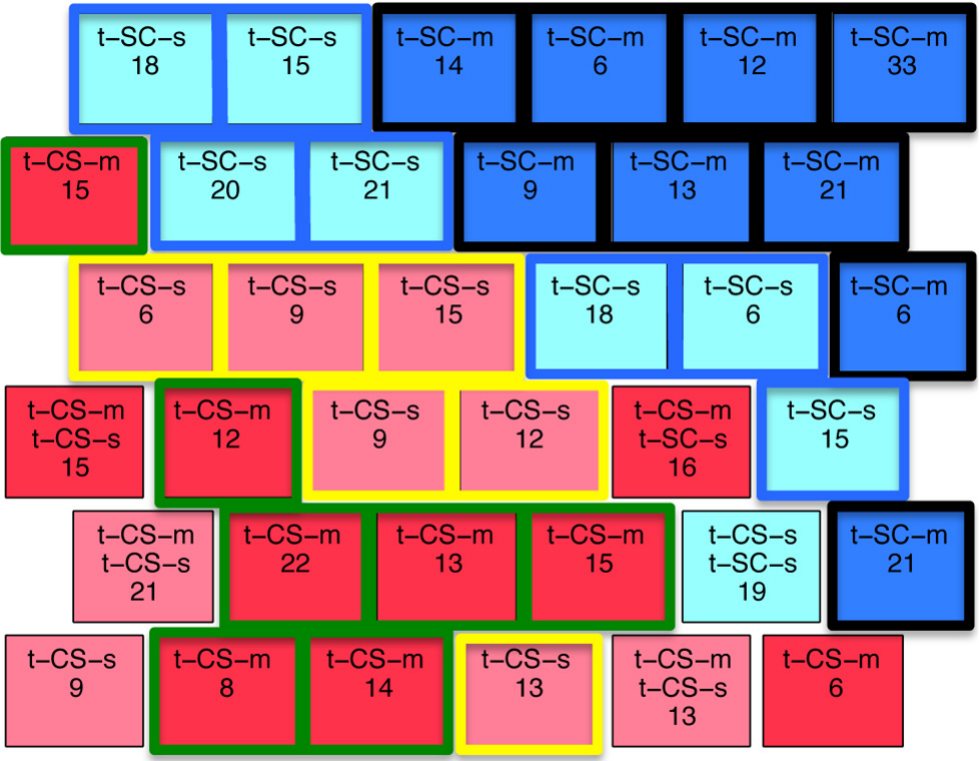
SOM clustering of trisomic mice data using 77 proteins. Light blue: t-SC-s; dark blue: t-SC-m; light pink: t-CS-s; dark pink: t-CS-m. Nodes forming each cluster are outlined: blue: t-SC-s; black: t-SC-m; yellow: t-CS-s; green: t-CS-m.

The optimal SOM for trisomic mice is shown in Fig 6 where the clusters of nodes have been outlined. Similar to Fig 2 with control mice, t-SC mice are well separated from t-CS (blue vs. pink). In addition, mice in t-SC-s and t-SC-m classes are also completely separated, with t-SC-s mice (light blue) in two clusters of 4 and 3 nodes, and t-SC-m (dark blue) in a large compact cluster of eight nodes and a single node. The organization of t-CS clusters, however, is more complicated and provides interesting differences from the SOM for control mice. In Fig 6, t-CS-s measurements are found in a cluster of 5 nodes (light pink) and one single node that together contain only 64 (of 105) measurements. Unlike control mice, the t-CS-s mice fail to learn and these nodes are largely located immediately adjacent to SC nodes, whereas in control mice, the c-CS-s nodes (Fig 2B) were distributed throughout the CS region. This difference suggests that protein levels in trisomy mice in failed learning are more similar to those in t-SC-s mice (i.e. in mice not asked to learn) than they are to those in control mice in successful learning. This is supported by the observation of mixed SC-CS nodes. Nodes containing the t-CS-m class (red background), the only class in this SOM that learns successfully, form a large 6-node cluster plus one singleton. Lastly, there are three CS-s/CS-m mixed nodes, containing 49 measurements, suggesting that not all responses in failed learning are incorrect and that these more closely resemble rescued learning.

#### i) Trisomic mice class-discriminant proteins

Comparison of t-CS-s vs. t-SC-s identifies differences in protein expression after the Ts65Dn mice have failed to learn in CFC. Using the Wilcoxon rank-sum test, ten proteins were found to be significantly different (S2 Table, c1): DYRK1A, ITSN1, pERK, BRAF, SOD1, MTOR, P38, NR2B, pP70S6, and GluR4. Six of these, BRAF, DYRK1A, ITSN1, P38, pERK and SOD1, also differed in the corresponding set of control mice in normal learning, c-CS-s vs. c-SC-s (Table 3, c1), again indicating that some normal responses occur even in failed learning. However, changes in an additional 25 proteins that occurred in normal learning in control mice were not seen in trisomy. Such a dramatic difference, in number and identity of protein responses, is consistent with failed learning.

Comparison of t-CS-m vs. t-SC-m identifies the proteins that respond when learning is rescued with memantine in trisomic mice. In contrast to failed learning, levels of 36 proteins are significantly different in this comparison (S2 Table, c2). Of these, seven were also seen in failed learning (S2 Table, c1) and a total of 17 responses also occurred in control mice in normal learning (proteins in italics in S2 Table, c2), suggesting that memantine acts to promote more, but still not all, normal responses to the stimulation of learning. A second comparison also reflecting learning, t-CS-m vs. t-SC-s, was made, identifying 20 proteins (S2 Table, c3; Table 3, column 4). Fig 3B clearly shows that relatively few changes occurred in failed learning and that, while memantine-rescued learning is associated with many more protein responses, only approximately half of these are the same as in control mice in normal learning. The Venn diagram in Fig 3B summarizes the number of proteins that are significantly different in these comparisons and their intersection among comparisons.

Because memantine facilitates learning in trisomy mice, it is reasonable to expect that changes produced by memantine alone, i.e. t-SC-m vs t-SC-s, include some that change the initial conditions to those more conducive to learning. Twelve proteins responded (S2 Table, c4): pNR2A, pPKCAB, pMTOR, pP70S6, pPKCG, S6, pS6, ARC, ERBB4, P3525, SNCA, and BAD. Only three were common to memantine in control mice (S1 Table, c5) and six also responded in normal learning (S1 Table, c1; Table 3, column 5).

The final comparison examines the differences in the end point protein profiles, t-CS-m vs. t-CS-s, i.e. compares the protein expression outcomes of rescued and failed learning. Only nine proteins were found (S2 Table, c4; Table 3, column 6): DYRK1A, pERK, BRAF, CDK5, RRP1, GFAP, GLUR3, P3525 and Ubiquitin.

#### ii) Validation of discriminant protein subsets

**Fig 7:**
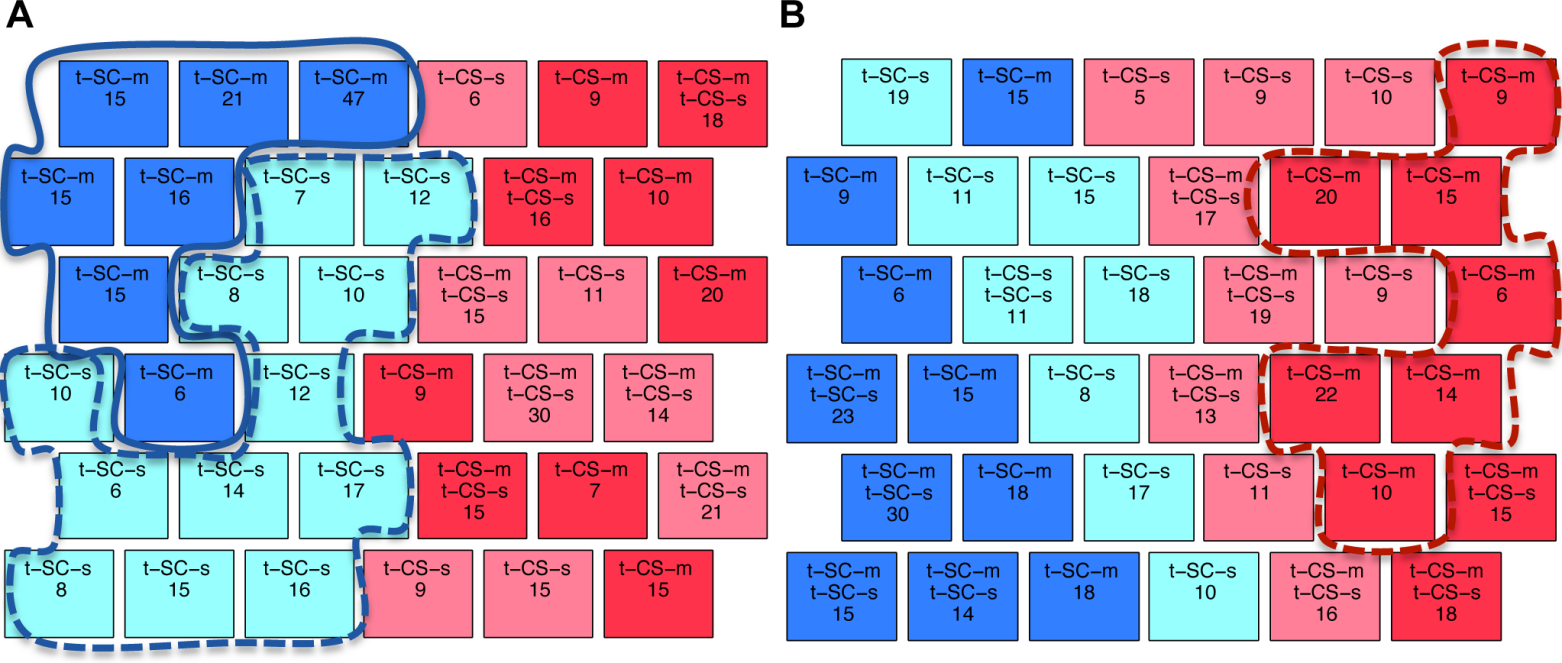
SOM clustering using subsets of proteins for trisomic mice. **(A)** Clustering of trisomic mice with proteins common to the two comparisons that reflected rescued learning: t-CS-m vs. t-SC-m and t-CS-m vs. t-SC-s (15 proteins), plus the initial effects of memantine: t-SC-m vs. t-SC-s (12 proteins). The t-SC-m cluster is outlined in solid blue and the t-SC-s cluster is outlined in dashed blue. **(B)** Clustering with the former 15 proteins (rescued learning) plus the 9 discriminant between t-CS-m and t-CS-s (S2 Table, c5).

To discover subsets of the 77 proteins that effectively discriminate classes of trisomic mice requires biological considerations different from those used for control mice. The Ts65Dn mice fail to learn when injected only with saline and therefore the comparisons of t-CS-s vs. t-SC-s and t-CS-s vs. t-SC-m do not reflect changes associated with successful learning. Instead of the four comparisons used with control mice, we considered first the protein changes that were common to the two comparisons that reflected rescued learning: t-CS-m vs. t-SC-m and t-CS-m vs. t-SC-s (15 proteins; Table 3, column 4), plus the initial effects of memantine: t-SC-m vs. t-SC-s (12 proteins; Table 3, column 5). The SOM generated with these proteins (a total of 26) is shown in Fig 7A. No nodes that mix CS and SC mice occur and all the measurements of the t-SC-s and t-SC-m classes are completely separated in two different clusters with no mixed class nodes. This distribution of the data is even better than that obtained with the 77 proteins, supporting the relevance of the subset identified. A second SOM, shown in Fig 7B, was generated using the 15 proteins that discriminate between rescued learning and the lack of stimulation to learn and the 9 proteins that discriminate between rescued learning and failed learning (Table 3, column 6) a total of 22 unique proteins. In this second clustering, there is a large cluster of t-CS-m nodes, containing 96 of 135 measurements of this type of mouse. There are also only five nodes containing solely t-CS-s mice, (containing 44 of 105 measurements). Instead, most appear mixed with measurements of t-CS-m mice.

### 3. Control versus trisomy mice SOM clustering and labeling

We used the same methods to identify proteins that best discriminate failed learning in trisomic mice from normal successful learning in control mice. Clustering using all eight groups of control and trisomic mice failed to produce a SOM with any clear clusters (data not shown). To search for an informative SOM, we then reduced the number of input classes to five: the three classes of successful learning (c-CS-s, c-CS-m, and t-CS-m), the one class of failed learning (t-CS-s) and one control class not stimulated to learn (c-SC-s). Together, these classes comprise 44 mice and 660 measurements that were clustered in a 7X7 SOM (S2 Fig). The control baseline mice, c-SC-s, were found in a single large cluster of ten adjacent nodes at the top of the SOM; none of the other four classes formed a large cluster and individual nodes of each class were interspersed throughout the map. Most interesting was the presence of 16 CS mixed nodes (of a total of 39 measurements), each containing measurements from 2, 3 or all 4 CS classes. While this indicates poor separation of the four classes, it is also biologically reasonable. Three classes learn successfully, and therefore similarities among subsets of their proteins are logical. Furthermore, from the trisomy SOMs described above, we also know that responses in the failed learning class do include a subset of the protein changes seen in successful learning. This latter is reflected in the presence of 10 of 16 mixed nodes that include measurements from t-CS-s mice plus one or more of the successful learning classes. Excluding the t-CS-s class from the clustering did not improve the clustering of successful learning classes (data not shown).

**Fig 8:**
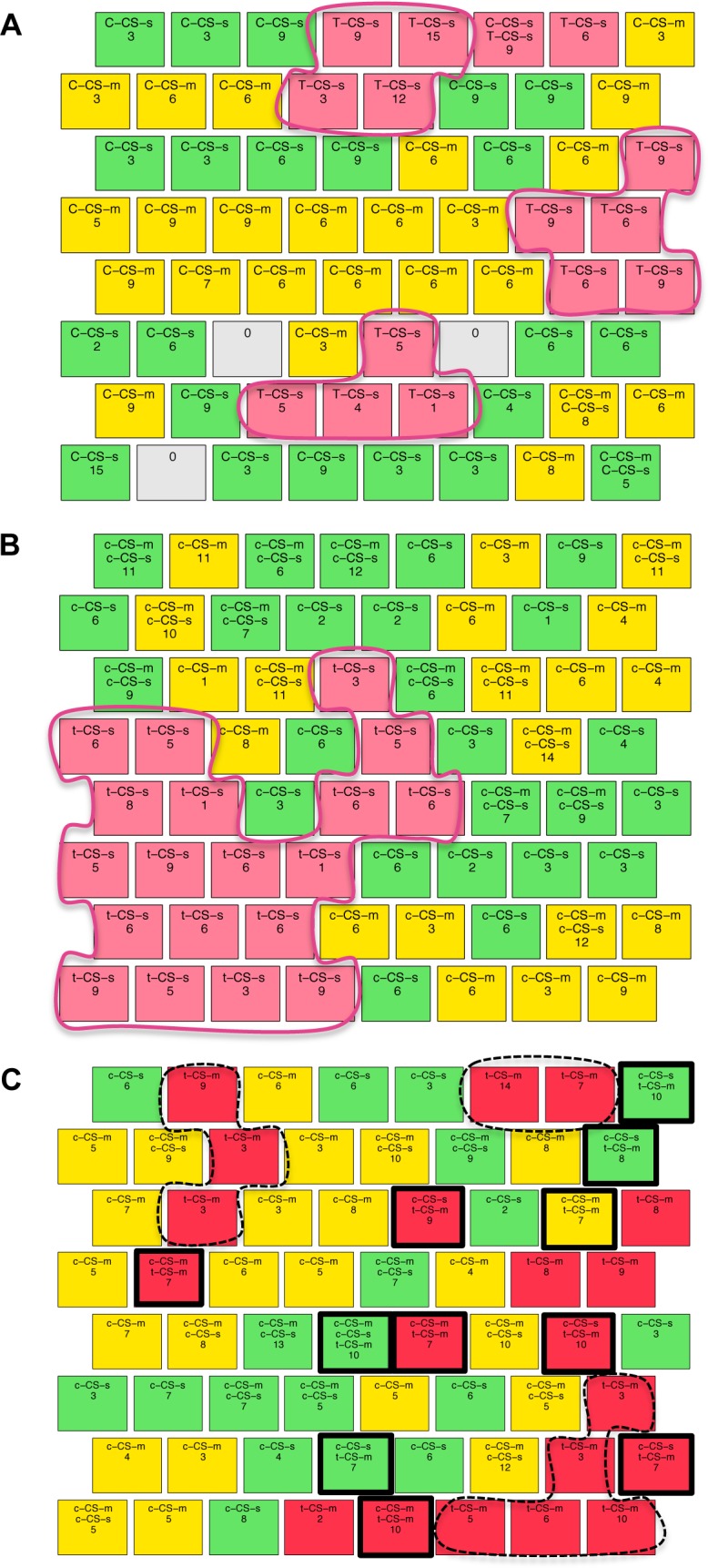
SOM clustering of CS classes of control and trisomic mice. **(A)** t-CS-s (failed learning, light pink nodes), c-CS-s (green) and c-CS-m (yellow), using as input the levels of all 77 proteins. Pink: clusters of t-CS-s. **(B)** t-CS-s, c-CS-s and c-CS-m using as input only the 10 proteins that discriminate t-CS-s from both c-CS-s and c-CS-m. Pink: clusters of t-CS-s. **(C)** t-CS-m (rescued learning, dark pink nodes), c-CS-s and c-CS-m using as input only the set of 10 proteins that discriminate t-CS-s from both c-CS-s and c-CS-m. Dashed black line: clusters of t-CS-m mice. Black squares: nodes with mixed classes of t-CS-m and controls.

We next clustered using t-CS-s, (failed learning) c-CS-s (normal learning) and c-CS-m (normal learning after memantine). These classes comprise 26 mice and 390 measurements. Using a 6x6 matrix produced poor separation of clusters (data not shown). We, therefore, chose to cluster with a SOM of 8x8 to allow clustering within a wider area. As shown in Fig 8A, t-CS-s mice are found in three clear clusters of 4–5 nodes containing 93 of the total of 105 measurements. Mice from the c-CS-s and c-CS-m are also each found in significant clusters, the largest containing ten and 14 nodes, respectively. The Wilcoxon rank-sum test identified 14 proteins significantly different between t-CS-s and c-CS-s (BDNF, pCAMKII, PKCA, pNR1, APP, MTOR, P38, AMPKA, NR2B, RAPTOR, S6, Tau, GluR3, Ubiquitin and EGR1) and 17 proteins differing in levels between t-CS-s with c-CS-m (NR2A, pNR1, APP, MTOR, P38, NR2B, RAPTOR, pGSK3B, S6, RRP1, BAX, nNOS, Tau, GFAP, GluR3, IL1B, EGR1). To validate these results, we performed clustering using the 10 proteins from the intersection of these two sets (underlined). In the resulting SOM (Fig 8B), the t-CS-s mice are found in a single large cluster completely separated from c-CS-s and c-CS-m. This suggests that the levels of these 10 proteins critically discriminate between a state incompatible with successful learning in this task in these Down syndrome mice and a state compatible with successful learning in control mice. As a further test, we used the same 10 proteins to cluster rescued learning (t-CS-m) with the two classes of successful learning in control mice. As shown in Fig 8C, the t-CS-m mice are dispersed throughout the SOM. There are four small clusters of 2–4 nodes, each containing only ~15–27 measurements. Indeed, 40% of the t-CS-m measurements are found in nodes mixed with c-CS-s or c-CS-m, and in one node with both. Together the SOMs in Fig 8 suggest that abnormal levels of these 10 proteins are critical to failed learning, and that memantine treatment induces changes in these responses that not only result in successful (rescued) learning, but also in a protein profile that is not distinguished from those of normal successful learning.

**Fig 9:**
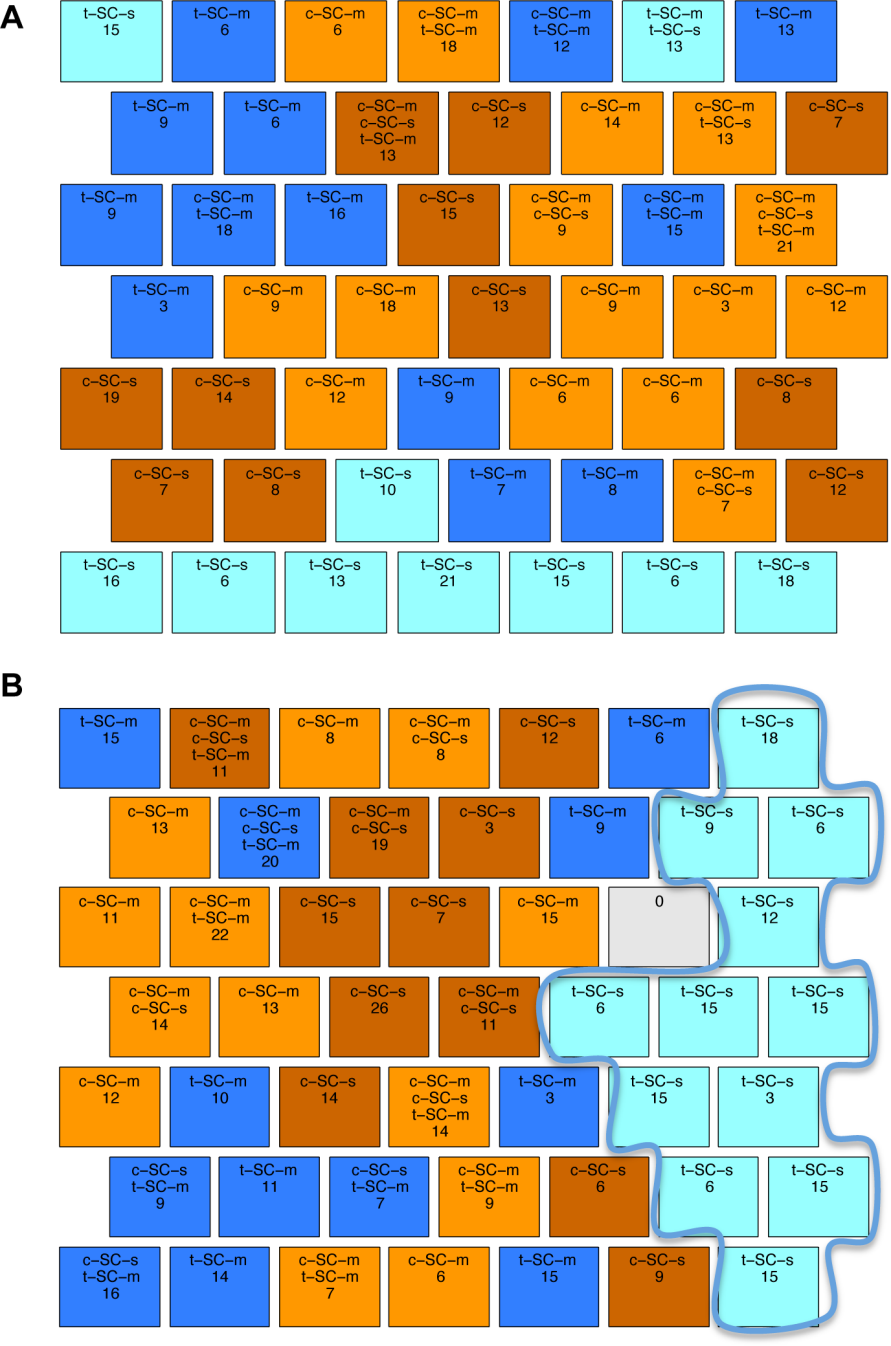
SOM clustering of shock-context classes of control and trisomic mice. **(A)** Clustering of classes: t-SC-s (light blue), t-SC-m (dark blue), c-SC-m (orange), c-SC-s (brown) with 77 proteins. **(B)** Clustering of the three classes with the 21 discriminant proteins found between t-SC-s and the two other classes.

The last clustering with control and trisomy mice used SC classes: t-SC-s, t-SC-m, c-SC-s and c-SC-m, i.e. the single initial state that is unable to promote successful learning, and the three initial states that can promote successful learning. Fig 9A shows the SOM obtained using all 77 proteins. The t-SC-s nodes are found in a single large cluster in the bottom row and one isolated node at the top of the map containing 15 measurements, i.e. almost completely separated from the other three classes. Of 135 measurements of t-SC-s, 120 are clustered in class specific nodes and only 15 are clustered with t-SC-m (11 t-SC-s measurements) or c-SC-m (4 t-SC-s measurements). We then calculated the discriminant proteins between t-SC-s and the other classes, identifying 21 discriminant proteins: pNR2A, pPKCAB, pRSK, APP, P38, pMTOR, pP70S6, pGSK3B, pPKCG, CDK5, S6, AcetylH3K9, ARC, Tau, IL1B, P3525, SNCA, Ubiquitin, pGSK3B_Tyr216, pS6 and CaNA. Fig 9B shows clustering with these 21 proteins. All t-SC-s mice are present in a single cluster of adjacent nodes separated from the rest of the classes. Also the appearance of an empty node at the border of the cluster reinforces the dissimilarity between the three classes and t-SC-s. These 21 proteins differentiate between trisomic mice that are incapable of learning successfully and the three classes of mice that are capable of learning with stimulation.

## Discussion

The molecular processes underlying learning and memory are among the most complex in mammalian systems. Mutation detection in humans has shown that several hundred genes can cause intellectual disability [4]. Genetic and pharmacological manipulations in mice have shown that several hundred additional genes can cause learning, memory and synaptic plasticity deficits. How these many genes normally work together to produce successful learning is largely unknown, yet it is an important problem to address. Intellectual disability with a genetic cause affects approximately 1% of the world population and pharmacotherapies are largely unknown [44]. DS represents a significant subpopulation of ID because it has an incidence of approximately one in 1000 live births worldwide [1]. DS is also particularly challenging because it is not due to a single gene mutation but to an extra copy of a normal chromosome that encodes ~160 protein coding genes of diverse functions, a small number of validated microRNA genes and a few hundred transcripts of undetermined functional properties [3]. An unknown subset of these genes are overexpressed due to copy number and it is hypothesized that this overexpression is sufficient to perturb normal pathways and normal responses to stimulation. Understanding the perturbations and the abnormal responses to stimulation is a logical step in understanding how drugs may rescue or be designed to rescue the abnormalities.

We have previously reported the measurement of 77 proteins in brains of control mice and the Ts65Dn partial trisomy mouse model of DS [10,16]. Measurements were made in a total of 8 groups of mice, controls and trisomy, with and without exposure to CFC, with and without treatment with the drug memantine. Because 15 measurements, composed of three replicates of a five point dilution series of each sample, were made for each protein in each mouse, a three-level mixed effects model [45] was used to determine the statistical significance of differences in protein levels between groups. After a Bonferroni correction for multiple testing, levels of 30%-40% of proteins were found to differ between any two groups. Because these proteins were diverse in their functions and not confined to components of one or two pathways or processes, the complexity is challenging to interpret. Furthermore, the analysis did not reveal which of the protein differences were most critical to any comparison, e.g. which of the protein differences contributed to the failure of the DS mice to learn or to rescue of learning when the mice were treated with memantine.

To address these types of questions, we developed a strategy using SOMs. Because SOMs project high dimensional data onto a 2-D space, they are well-suited for discovery of patterns hidden in the input data. SOMs were used here to cluster expression data using both the complete set of 77 proteins/features and subsets of them. We used as input, data from different combinations of the eight groups of mice, where the choice of groups was driven by the biological questions of interest. First, we examined SOMs generated only with groups of control mice to evaluate molecular events in normal learning; we then examined SOMs with the corresponding groups of trisomic mice, to examine failed learning and its rescue by memantine; and finally we generated SOMs with combinations of control and trisomic groups to examine differences in learning caused by trisomy. In each case, after clustering, nodes in the SOM were labeled with the name(s) of the class(es) of mice from which the data contained in them were derived and the total number of measurements they contained. Color coding of nodes by class content allows visualization of the distributions of single-group nodes, the sizes of clusters of single-group nodes, and the number and distribution of mixed-group nodes.

The first SOM was generated using the complete dataset of 77 proteins from the four groups of control mice (Fig 2). In this SOM, c-CS mice were clearly separated from c-SC mice and there were no nodes containing both CS and SC measurements, thus showing that differences in the levels of these proteins discriminate successful learning from the lack of stimulation to learn. This outcome was consistent with the number of proteins (25 and 31, respectively) previously determined to differ significantly between learning and no learning in control mice, with and without memantine [10]. Next, we considered the four comparisons associated with successful learning, c-CS-s/m vs. c-SC-s/m, and used the Wilcoxon-rank sum test to determine the set of proteins that differed significantly in each of the four comparisons. Common to all four comparisons were differences in 11 proteins (Fig 3A; Table 3 column 1). The possibility that these proteins are critical to successful learning was tested by generating an SOM using only these 11 proteins (Fig 4A). The separation of CS from SC mice was maintained, i.e., these proteins were sufficient for discriminating successful normal learning from no stimulation to learn. In addition, the number of mixed CS-s plus CS-m nodes doubled, as did the number of mixed SC-s and SC-m nodes. The increases in numbers of mixed-nodes support the relevance of these 11 proteins for discriminating CS vs. SC rather than discriminating saline vs. memantine. Lastly, clustering with the remaining 66 proteins produced a SOM in which the separation between CS and SC was completely lost, suggesting that the 11 proteins are necessary for successful clustering/discrimination. Together these results strongly support the relative biological importance to learning of the 11 proteins.

A similar application of SOM to the protein dataset from the trisomic mice produced very different results. Unlike control c-CS-s mice, trisomic t-CS-s mice fail to learn in CFC. Consistent with this, when the four groups of trisomic mice were clustered, the SOM showed a t-CS-s cluster of nodes immediately adjacent to the t-SC nodes, plus two nodes that mixed CS and SC measurements (Fig 6). In addition, in the trisomic SOM, only 15% of CS nodes contained both CS-s and CS-m measurements, while in control mice, 30% of CS nodes were mixed c-CS-s and c-CS-m. Together, these SOM characteristics indicate that protein responses when trisomic mice fail to learn in CFC more closely resemble responses in mice that were not stimulated to learn than do control mice after successful learning. Consistent with this, of the ten proteins that changed significantly in failed learning, only five were common to the subset of 11 critical proteins in control mice successful learning. These are logical reflections of the failure to learn.

The analysis of SOMs using data from control and trisomic mice as input features illustrated this concept further. A common set of ten proteins were identified that discriminated between t-CS-s and both c-CS-s and c-CS-m, i.e. between failed learning in trisomic mice and successful learning in control mice (Table 3 column 7). Changes, or the lack thereof, in the levels of these proteins in failed learning, were therefore either inadequate to promote, or inhibitory to, learning.

In Table 3, we list the proteins that discriminate between groups in eight of the comparisons that were used in Figs 4, 5 and 7–9. These comparisons include successful learning in controls (normal learning), rescued and failed learning in trisomics, and memantine effects on initial (SC) and endpoint (CS) conditions in controls and trisomics. Proteins are organized in eight functional categories (plus a miscellaneous group). Methods for assessing functional enrichment, e.g., based on Gene Ontology (GO) terms, tools in DAVID or Gene Set Enrichment Analysis [46–48], are not appropriate here. Such methods were designed for use with large lists of genes, typically from whole genomes and oligonucleotide arrays, where sequences or probes for tens of thousands of genes, essentially an unbiased, complete transcriptome, are interrogated. In contrast, the proteins we measured by RPPA do not provide an unbiased interrogation of the proteome. On the contrary, they are heavily biased, specifically to those proteins that are known to function in learning and memory, in synaptic plasticity or synaptic transmission and/or have been observed to be abnormal in level in brains from people with DS or mouse models of DS [16]. The eight functional classes in Table 3 include both the MAP kinase and MTOR pathways, and immediate early genes (IEGs). Components of these are well established by genetic and pharmacological studies to be critical for normal L/M [41,42,49–51]. Similarly, those associated with Alzheimer’s Disease and the NMDAR have well established roles in synaptic plasticity and synaptic transmission, as discussed previously [9]. Memantine is an antagonist of the NMDA receptor, which is predicted to be hyper activated in AD and in DS [12,13], [15]. Therefore, NMDAR subunits, NR1, NR2A and NR2B, and their protein interaction partners, including GLUR3 and APP, would reasonably be predicted to be impacted. Also included in several columns in Table 3 is P38 that signals downstream of the NMDAR [52]. However, it must be noted that the remaining RPPA proteins, those excluded from Table 3 because they did not discriminate between the groups listed, also largely belong to these same functional categories. For example, 20 and 14 protein components of the MAPK and MTOR pathways were measured [10,16], but only nine in each pathway differed in any comparison in Table 3, and only two and four components of the MAPK pathway differed, respectively, in normal learning and rescued learning. The discovery here then is not the importance to learning and memory of the functional classes in Table 3, because these are already well established. Rather, it is the identification, within these pathways and processes, of the specific components that are critical to normal learning, to its disruption in this DS mouse model, and to its rescue by the drug memantine.

In Table 3, columns 1 and 4 list the proteins that are discriminating in normal learning and rescued learning, respectively. Two components of the MTOR pathway, S6 and phosphoGSK3B-S9, are specific to normal learning and two different MTOR pathway components, MTOR and AKT, are specific to rescued learning. Common to both normal and rescued learning are two components of the MAPK pathway, BRAF and the phosphorylated form of ERK. The latter has been well documented for playing a pivotal role, not only in learning and memory in general, but in CFC in particular [53,54]. That pERK is selected among 77 proteins for a critical role supports the validity of the SOM approach.

Because the focus of this work is on DS, it is particularly interesting that three proteins encoded by Hsa21 are included in both normal and rescued learning. DYRK1A is a protein kinase that includes among its substrates transcription factors, RNA processing enzymes, and proteins involved in endocytosis and the cytoskeleton [55,56]. ITSN1 is a multiple domain protein that functions in endocytosis, and in brain, via a neuronal-specific isoform, encodes a guanine nucleotide exchange factor with activity for CDC42 [57]. SOD1, superoxide dismutase, contributes to elevated levels of reactive oxygen species seen in DS [58]. While knockouts of DYRK1A and ITSN1 lead to neurological abnormalities [59–61] and overexpression of DYRK1A and of SOD1 in transgenic mice leads to learning and memory deficits [56], the experiments here are the first to show their responses directly in normal learning. All three Hsa21 proteins, DYRK1A, ITSN1 and SOD1, are overexpressed in the Ts65Dn mice analyzed here [16]. It is of interest therefore that levels increase further in rescued learning.

We emphasize the importance of this set of proteins to brain function by considering their associations with 13 pathways well established for roles in learning/memory and/or brain development, structure or function. Table 6 lists these pathways plus the number of components that are either RPPA proteins or that interact with one or more RPPA proteins from Table 3. For example, more than 20% of components of the MTOR and long term potentiation pathways are Table 3 proteins, and 70% and 55% of components of these pathways furthermore interact with one or more of this subset of these RPPA proteins. For the majority of pathways in Table 6, 10%-25% of components are in Table 3 and on average >40% of pathway components interact with Table 3 proteins.

**Table 6:** SOM discriminating proteins are enriched in pathways relevant to neurological function.

KEGG Pathway	#	RPPA components	%	# with RPPA PPI	%
mTOR signaling pathway	61	17	28	43	70
Long-term potentiation	70	17	24	39	56
ErbB signaling pathway	88	18	20	63	72
Alzheimer's disease	50	8	16	29	58
HIF-1 signaling pathway	109	16	15	56	51
Neurotrophin signaling pathway	116	16	14	69	59
Cholinergic synapse	95	12	13	30	32
Dopaminergic synapse	124	14	11	49	40
Glutamatergic synapse	101	10	10	31	31
Apoptosis	86	7	8	44	51
Serotonergic synapse	87	7	8	21	24
MAPK signaling pathway	256	18	7	122	48
Axon guidance	118	6	5	33	28

Pathways were obtained from the Kyoto Encyclopedia of Genes and Genomes database http://www.kegg.jp/. Proteins listed in Table 3 were used to search the KEGG pathway database. #, total number of protein components in the pathway; RPPA component, %: number/% of proteins from Table 3 that are components of the pathway; # with RPPA PPI/%: number/% of pathway components that interact with one or more proteins from Table 3.

The application of SOM to complex protein profiles has provided a novel look at the molecular consequences of normal and failed learning. These data, and conclusions, are far from complete however, and require further study. First, additional subcellular fractions and brain regions must be analyzed. Successful learning in CFC requires a functional hippocampus and it is hippocampus that has been studied most often at the molecular level. Protein levels in other brain regions are clearly dynamic, however, and undoubtedly important [62]. For this initial analysis, we chose to analyze the data from the nuclear-enriched fraction of cortex because the dataset was the most complete. It remains important to extend this type of analysis to the datasets from the cytosol and membrane fractions of cortex, and to all three fractions of the hippocampus. The complexity of such an analysis will be significantly increased because relationships among responses in different brain regions and fractions will also need to be considered. Second, only a single time point after training was assessed. Following the time course of responses will provide better understanding of exactly what completely fails to occur in the trisomic mice vs. what merely fails to occur in the properly orchestrated time frame. Third, this analysis compared static protein levels; dynamic responses, i.e. a change in level of some proteins, regardless of the initial or final level, could be critical for learning to occur, and their absence in DS could contribute to failures to learn. Data from current experiments could be analyzed for such contributions. Expansion in the complexity of the datasets will likely require consideration of computational techniques in addition to SOM. Lastly, while each protein was selected for its known role in brain development, or learning, memory or synaptic plasticity, there are many other proteins of interest that were not measured. Reverse Phase Protein Arrays that were used for protein measurement require highly specific antibodies and these are not available for some proteins of interest. The SOMs generated here therefore are affected by the specific sets of proteins used as input, and measurement of different proteins could change the discriminating sets.

Further validation of the observations regarding normal learning in control mice will benefit from repeating CFC studies with mice from different genetic background strains [63]. It is well known that the same mutation present on different inbred backgrounds can give rise to very different phenotypic features, in some cases eliminating any observable consequence of the mutation. It will be of interest to generate SOMs with data from several genetic backgrounds where learning is normal in CFC. This might serve to identify new classes of critical proteins. For trisomic mice, important data would be protein responses after treatment with other drugs that also rescue learning in CFC. Currently there are more than half a dozen such drugs and they are diverse in their known targets and mechanisms of action, and thus are expected to also be diverse in at least some of their protein responses [64]. SOMs could be used to identify common critical protein responses which would help to define potentially more effective targets. Additional experiments with other mouse models of DS, models that are trisomic for different sets of orthologs of Hsa21 genes, will also provide useful information to understand the set of perturbations that might arise in people with DS who are trisomic for all the genes encoded by Hsa21.

## Supporting Information

S1 Fig Levels of Wilcoxon test p-values. Levels of 12 proteins with the lowest p-values of Wilcoxon test discriminating c-CS-s and c-SC-s clusters.
(EPS)

S2 Fig SOM clustering with the 77 proteins of the four context-shock classes of control and trisomic mice and one shock-context control class. Clustering of classes c-CS-s (green nodes), c-CS-m (yellow), t-CS-s (light pink), t-CS-m (dark pink) and c-SC-s (brown).
(TIF)

S1 Table Discriminant proteins found in comparisons of control mice classes. (DOCX)

S2 Table Discriminant proteins found in comparisons of trisomic mice classes. (DOCX)

S1 Dataset Protein expression levels of 77 proteins measured in the nuclear fraction of cortex from control and Down syndrome mice (Ts65Dn). These data are a subset of those used in Ahmed et al PLoS ONE 2015 10(3): e0119491
(ZIP)
