# Supplementary figures and images for: Self-Organizing Feature Maps Identify Proteins Critical to Learning in a Mouse Model of Down Syndrome
Source: PLoS One. 2015 Jun 25;10(6):e0129126. doi: 10.1371/journal.pone.0129126 (PMC4482027; doi:10.1371/journal.pone.0129126)

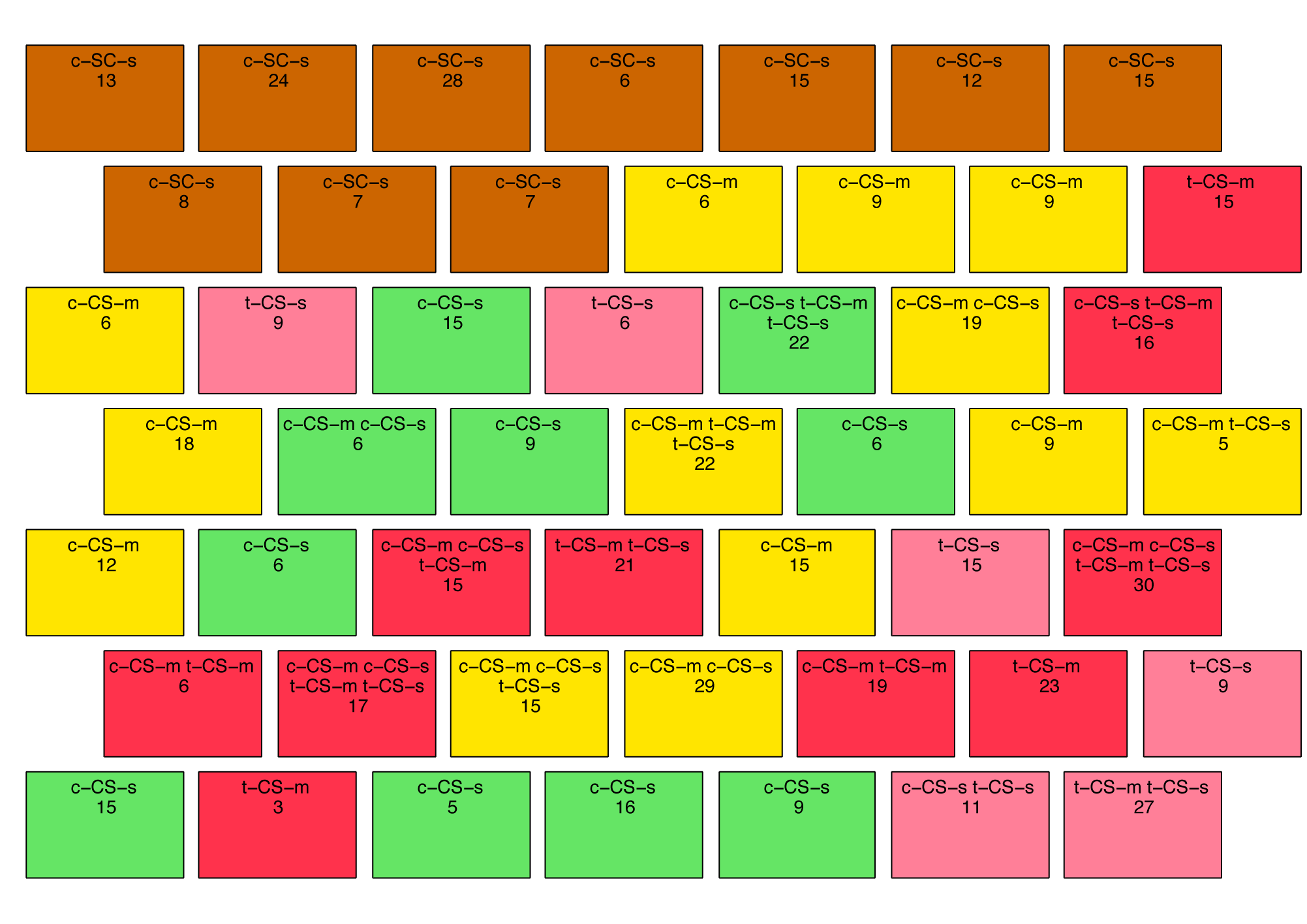

Supplement: S2 Fig — Clustering of classes c-CS-s (green nodes), c-CS-m (yellow), t-CS-s (light pink), t-CS-m (dark pink) and c-SC-s (brown). (TIF) [file pone.0129126.s002.tif]
