# Supplementary material for: Self-Organizing Feature Maps Identify Proteins Critical to Learning in a Mouse Model of Down Syndrome
Source: PLoS One. 2015 Jun 25;10(6):e0129126. doi: 10.1371/journal.pone.0129126 (PMC4482027; doi:10.1371/journal.pone.0129126)
Supplement: S1 Table — (DOCX) [file pone.0129126.s003.docx]

**Table S1: Discriminant proteins found in comparisons of control mice classes.**

| **c1** | **c2** | **c3** | **c4** | **c5** | **c6** |
| --- | --- | --- | --- | --- | --- |
| **c-CS-s vs. c-SC-s** | **c-CS-m vs. c-SC-m** | **c-CS-m vs. c-SC-s** | **c-CS-s vs. c-SC-m** | **c-SC-m vs. c-SC-s** | **c-CS-m vs. c-CS-s** |
| **APP** | ADARB1 | BRAF | AMPKA | AKT | ARC |
| ARC | **APP** | CaNA | APP | ARC | BAD |
| BAD | **BDNF** | CDK5 | ARC | BCL2 | EGR1 |
| **BDNF** | **BRAF** | DYRK1A | BAD | ELK | ERBB4 |
| **BRAF** | **CaNA** | GFAP | Bcatenin | H3AcK18 | H3MeK4 |
| **CaNA** | **CDK5** | ITSN1 | BCL2 | NR1 | IL1B |
| **CDK5** | **DYRK1A** | pERK | BDNF | pCAMKII | nNOS |
| **DYRK1A** | **EGR1** | pGSK3B | BRAF | pNR1 | PKCA |
| **EGR1** | **ERK** | pGSK3B_Tyr216 | CaNA | pNUMB | pPKCAB |
| ERBB4 | **GFAP** | pNUMB | CDK5 | pPKCG | pS6 |
| **ERK** | **GSK3B** | pP70S6 | DYRK1A | pS6 | SHH |
| **GFAP** | **ITSN1** | S6 | EGR1 | SOD1 | Ubiquitin |
| **GSK3B** | NR2A | SOD1 | ELK | Ubiquitin |  |
| H3MeK4 | P3525 |  | ERK |  |  |
| IL1B | **P38** |  | GFAP |  |  |
| **ITSN1** | pCAMKII |  | GSK3B |  |  |
| nNOS | **pERK** |  | H3AcK18 |  |  |
| **P38** | **pGSK3B** |  | H3MeK4 |  |  |
| **pERK** | **pNUMB** |  | IL1B |  |  |
| **pGSK3B** | **S6** |  | ITSN1 |  |  |
| PKCA | **SOD1** |  | nNOS |  |  |
| **pNUMB** | TRKA |  | NR1 |  |  |
| pPKCAB | **Ubiquitin** |  | NR2A |  |  |
| pRSK |  |  | NUMB |  |  |
| pS6 |  |  | P38 |  |  |
| PSD95 |  |  | pCAMKII |  |  |
| **S6** |  |  | pCFOS |  |  |
| SHH |  |  | pERK |  |  |
| SNCA |  |  | pGSK3B |  |  |
| **SOD1** |  |  | PKCA |  |  |
| **Ubiquitin** |  |  | pNUMB |  |  |
|  | |  | pPKCAB |  |  |
|  | |  | pS6 |  |  |
|  |  |  | PSD95 |  |  |
|  |  |  | S6 |  |  |
|  |  |  | SNCA |  |  |
|  |  |  | SOD1 |  |  |
|  |  |  | TRKA |  |  |
|  |  |  | Ubiquitin |  |  |

Bold indicates common proteins between c1 and c2.
