# Supplementary material for: Self-Organizing Feature Maps Identify Proteins Critical to Learning in a Mouse Model of Down Syndrome
Source: PLoS One. 2015 Jun 25;10(6):e0129126. doi: 10.1371/journal.pone.0129126 (PMC4482027; doi:10.1371/journal.pone.0129126)
Supplement: S2 Table — (DOCX) [file pone.0129126.s004.docx]

**Table S2: Discriminant proteins found in comparisons of trisomic mice classes**

| **c1 (FL)** | **c2 (RL)** | **c3** | **c4** | **c5** |
| --- | --- | --- | --- | --- |
| **t-CS-s vs. t-SC-s** | **t-CS-m vs. t-SC-m** | **t-CS-m vs- t-SC-s** | **t-SC-m vs. t-SC-s** | **t-CS-m vs. t-CS-s** |
| **BRAF** | AKT | AcetylH3K9 | **ARC** | **BRAF** |
| **DYRK1A** | AMPKA | AKT* | BAD | CDK5 |
| GluR4 | ***ARC*** | BAD | **ERBB4** | **DYRK1A** |
| **ITSN1** | ***BRAF*** | BRAF* | **P3525** | GFAP |
| **MTOR** | CAMKII | CaNA* | **pMTOR** | GluR3 |
| NR2B | *CaNA* | DYRK1A* | **pNR2A** | **P3525** |
| **P38** | DSCR1 | EGR1* | **pP70S6** | **pERK** |
| **pERK** | ***DYRK1A*** | H3AcK18* | pPKCAB | RRP1 |
| **pP70S6** | *EGR1* | H3MeK4* | pPKCG | **Ubiquitin** |
| **SOD1** | ***ERBB4*** | ITSN1* | **pS6** |  |
|  | *GSK3B* | MTOR* | **S6** |  |
|  | H3AcK18 | P38* | SNCA |  |
|  | *H3MeK4* | pERK* |  |  |
|  | ***ITSN1*** | pMEK* |  |  |
|  | **MTOR** | pP70S6 |  |  |
|  | NR2B | RRP1 |  |  |
|  | ***P38*** | SNCA* |  |  |
|  | pAKT | SOD1* |  |  |
|  | pBRAF | Tau |  |  |
|  | pCREB | Ubiquitin* |  |  |
|  | ***pERK*** |  |  |  |
|  | *pGSK3B* |  |  |  |
|  | pGSK3B_Tyr216 |  |  |  |
|  | pJNK |  |  |  |
|  | pMEK |  |  |  |
|  | **pMTOR** |  |  |  |
|  | **pNR2A** |  |  |  |
|  | pNR2B |  |  |  |
|  | ***pS6*** |  |  |  |
|  | RAPTOR |  |  |  |
|  | RSK |  |  |  |
|  | ***S6*** |  |  |  |
|  | *SNCA* |  |  |  |
|  | ***SOD1*** |  |  |  |
|  | TIAM1 |  |  |  |
|  | ***Ubiquitin*** |  |  |  |

Bold indicates proteins that responded in more than one comparison.

Italics indicate proteins that also changed in control mice in normal learning (c-CS-s vs. c-SC-s; Table 2 c1).

* indicates proteins common to comparisons of c2 and c3.
